# *Bos taurus *genome assembly

**DOI:** 10.1186/1471-2164-10-180

**Published:** 2009-04-24

**Authors:** Yue Liu, Xiang Qin, Xing-Zhi Henry Song, Huaiyang Jiang, Yufeng Shen, K James Durbin, Sigbjørn Lien, Matthew Peter Kent, Marte Sodeland, Yanru Ren, Lan Zhang, Erica Sodergren, Paul Havlak, Kim C Worley, George M Weinstock, Richard A Gibbs

**Affiliations:** 1 Human Genome Sequencing Center, Department of Molecular and Human Genetics, Baylor College of Medicine, One Baylor Plaza, Houston, TX, 77030, USA; 2 Department of Computer Science and Center for Computational Biology and Bioinformatics, Columbia University, New York, NY, USA; 3 Department of Biomolecular Engineering, University of California at Santa Cruz, Santa Cruz, CA, 95064, USA; 4 Centre for Integrative Genetics and Department of Animal and Aquacultural Sciences, Norwegian University of Life Sciences, Arboretveien 6, Ås, 1432, Norway; 5 Washington University St Louis, MO, USA; 6 Department of Computer Science, University of Houston, 4800 Calhoun Road, Houston, TX 77204-3010, USA

## Abstract

**Background:**

We present here the assembly of the bovine genome. The assembly method combines the BAC plus WGS local assembly used for the rat and sea urchin with the whole genome shotgun (WGS) only assembly used for many other animal genomes including the rhesus macaque.

**Results:**

The assembly process consisted of multiple phases: First, BACs were assembled with BAC generated sequence, then subsequently in combination with the individual overlapping WGS reads. Different assembly parameters were tested to separately optimize the performance for each BAC assembly of the BAC and WGS reads. In parallel, a second assembly was produced using only the WGS sequences and a global whole genome assembly method. The two assemblies were combined to create a more complete genome representation that retained the high quality BAC-based local assembly information, but with gaps between BACs filled in with the WGS-only assembly. Finally, the entire assembly was placed on chromosomes using the available map information.

Over 90% of the assembly is now placed on chromosomes. The estimated genome size is 2.87 Gb which represents a high degree of completeness, with 95% of the available EST sequences found in assembled contigs. The quality of the assembly was evaluated by comparison to 73 finished BACs, where the draft assembly covers between 92.5 and 100% (average 98.5%) of the finished BACs. The assembly contigs and scaffolds align linearly to the finished BACs, suggesting that misassemblies are rare. Genotyping and genetic mapping of 17,482 SNPs revealed that more than 99.2% were correctly positioned within the Btau_4.0 assembly, confirming the accuracy of the assembly.

**Conclusion:**

The biological analysis of this bovine genome assembly is being published, and the sequence data is available to support future bovine research.

## Background

Genome assembly, the process of combining short sequences to represent a consensus sequence of a genome, is always a compromise. Assembly methods are chosen that can be applied to the entire genome for a consistent result. The sequences can be aggressively merged creating false joins in some cases but at the same time producing a statistically more contiguous assembly. Or, sequences can be conservatively merged, leaving many contigs and scaffolds unjoined but creating fewer false joins. Random sequences assemble more consistently than genomic sequence where the complications are due to the non-random nature of genomic sequence, such as repetitive sequences and polymorphisms.

There have been few main methods used for genome assembly. The human genome[1] was assembled using a hierarchical approach, where bacterial artificial chromosomes (BACs) were isolated and mapped to the genome and then individually sequenced. The advantage of this method is that the individual BACs contain a single haplotype and the assembly within a BAC avoids conflicts due to polymorphisms and as a result is more contiguous and correct for a given level of sequence coverage. The main disadvantage of this method is the cost associated with mapping the BACs and generating individual sequence libraries for each BAC.

To reduce the cost of BAC cloning and library construction, the whole genome shotgun (WGS) method has been used for a number of genomes. While there are many advantages to the method, a WGS approach has difficulties dealing with repetitive sequences in the genome that tend to collapse in assembly, and in resolving regions of polymorphisms between the two haplotypes in the genome that may be sufficiently different to assemble as two copies rather than as one. The first mouse genome used the WGS approach, as did the macaque[2], dog[3], opossum[4], platypus, chimpanzee[5] and the low coverage genome sequences including cat[6].

Some of these assemblies benefited from comparison to closely related species to improve the assembly. While this is a powerful approach, it can hide true differences between the species that are only seen in the new genome.

The bovine genome sequence reported here, like the rat genome sequence[7] employed a combined WGS plus BAC approach. Like the sea urchin[8], many of the BACs for the bovine project were sequenced in pools rather than individually, as a cost saving measure. In contrast to previous assemblies, the bovine assembly leveraged the benefit of local assembly provided by the BACs by tuning the assembly parameters for each BAC to address local differences in sequence characteristics (e.g. repeat content and degree of polymorphism compared to the WGS sequence) to produce the best assembly within each enriched BAC (eBAC).

The bovine project was fortunate to have many sets of markers from different sources available to place the assembly on chromosomes. A challenge in using these was the difficulty in merging the multiple marker sets into a single consistent map. New software (Atlas) assembly components were developed to solve the conflicts in the merged marker sets and maximize their usage for scaffold placement and correction.

## Results

The bovine genome was assembled at the Baylor College of Medicine Human Genome Sequencing Center using a combined method similar to that used for the rat genome[9] and more recently the sea urchin genome[8]. The combined strategy is a hybrid of the Whole Genome Shotgun (WGS) approach used for the mouse genome and the hierarchical (BAC clone) approach used for the human genome. The sequencing combines BAC shotgun reads with whole-genome-shotgun (WGS) reads from small insert libraries as well as BAC end sequences (BES).

The DNA for the small insert WGS libraries was from white blood cells from the Hereford cow L1 Dominette 01449. The source of the BAC library DNA was Hereford bull L1 Domino 99375, the sire of the former animal.

Two early assembly versions (Btau_1.0 and Btau_2.0) were prepared using only whole genome shotgun (WGS) reads from small insert clones and BES. Contigs from Btau_2.0 were used in the subsequent assembly.

**Figure 1:**
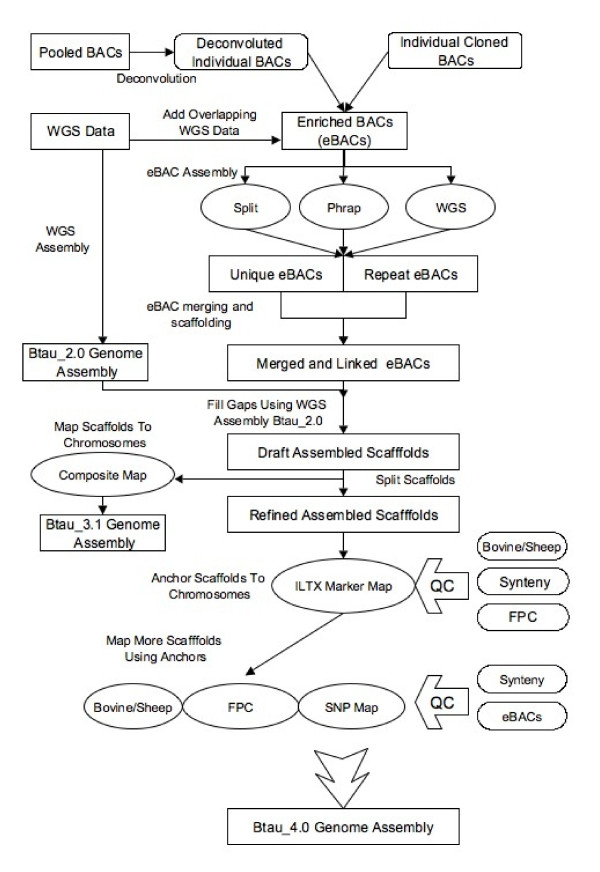
**The Genome Assembly process**. Sequence from pooled BACs, individual BACs and Whole Genome Shotgun was combined in a number of different ways as outlined here. At the top left, pooled BACs were deconvoluted and assembled as individual BACs. On the top right, individually sequenced BACs were also assembled as individual BACs. Overlapping WGS data was added to all BACs and each was assembled as an enriched BAC (eBAC) using three different assembly methods. The best assembly of each eBAC was used in the merging and scaffolding steps. On the left, WGS data was assembled as a WGS assembly to produce Btau_2.0. Contigs from this assembly that were not contained within the eBACs were used to fill gaps in the BAC assembly. The sequence scaffolds were placed on chromosomes using the composite map (Integrated Bovine Map) to produce Btau_3.1. Some scaffolds were split and a multi-step placement procedure described in the text was used to place scaffolds on chromosomes for version Btau_4.0. A more complete description can be found in the text.

Btau_3.1 was produced using the Atlas genome assembly system with a combination of WGS and BAC sequence[10]. The assembly process consisted of multiple phases (Figure 1). Sequences from each BAC were assembled with Phrap, first with just the BAC generated sequences, then in combination with the WGS reads that overlapped the BAC as an enriched BAC (eBAC). BACs were sequenced as either individual clone libraries or as pools of arrayed clones (see read statistics in Table 1 and basepair statistics in Table 2). BAC reads from individual libraries or from deconvoluted pools were assembled as individual BACs. 19,667 BAC projects (12,549 individual sequenced clones and 7,118 clones from BAC pools) were sequenced and assembled. Details of BAC assembly methods are provided below. Contigs from the Btau_2.0 WGS assembly were used to fill in the gaps in the BAC-based assembly (e.g. those due to gaps in the BAC tiling path), creating the combined assembly, Btau_3.1.

**Table 1:** Read Statistics

	**Btau 1.0**		**Btau 2.0**		**Btau 3.1**		**Btau 4.0**	
**InsertSize(kb)**	**2 to 4**	**4 to 6**	**2 to 4**	**4 to 6**	**2 to 6**	**200**	**2 to 6**	**200**
**Source/Vector**	**Plasmid**	**Plasmid**	**Plasmid**	**Plasmid**	**Plasmid**	**BAC**	**Plasmid**	**BAC**
**Reads **(million)								
**WGS**								
All WGS	14.09	1.41	NA	NA	26.98	0.21	26.98	0.21
Total	15.51		27.2		27.19		27.19	
Trimmed	11.72	1.15	19	4	23.09	0.17	23.09	0.17
Total	12.88		23		23.26		23.26	
Paired	11.01	1.07	5	5.2	NA	NA	NA	NA
Total	12.08		10.2		NA		NA	
Assembled	8.98	0.89	14.9	3.2	18.74	0.11	18.74	0.11
Total	9.87		18.1		18.86		18.86	
Unassembled	0.39	0.04	0.31	0.06	NA	NA	NA	NA
Total	0.43		0.37		NA		NA	
**Single BACs**								
All	NA	NA	NA	NA	6.42	NA	6.42	NA
Total	NA		NA		6.42		6.42	
Trimmed	NA	NA	NA	NA	5.11	NA	5.11	NA
Total	NA		NA		5.11		5.11	
Assembled	NA	NA	NA	NA	4.28	NA	4.28	NA
Total	NA		NA		4.28		4.28	
**Pooled BACs**								
All	NA	NA	NA	NA	5.38	NA	5.38	NA
Total	NA		NA		5.38		5.38	
Trimmed	NA	NA	NA	NA	5.12	NA	5.12	NA
Total	NA		NA		5.12		5.12	
Assembled	NA	NA	NA	NA	2.92	NA	2.92	NA
Total	NA		NA		2.92		2.92	

**Table 2:** Basepair Statistics

	**Btau 1.0**		**Btau 2.0**		**Btau 3.1**		**Btau 4.0**	
**InsertSize(kb)**	**2 to 4**	**4 to 6**	**2 to 4**	**4 to 6**	**2 to 6**	**200**	**2 to 6**	**200**
**Source/Vector**	**Plasmid**	**Plasmid**	**Plasmid**	**Plasmid**	**Plasmid**	**BAC**	**Plasmid**	**BAC**
**Bases **(billion)								
Trimmed	8.34	0.84	14.5	3.2	24.2	0.1	24.2	0.1
Total	9.18		17.7		24.3		24.3	
Asssembled	6.48	0.65	11.6	2.5	19	0.1	19	0.1
Total	7.13		14.1		19.1		19.1	
Unassembled	0.2	0.023	0.13	0.04	N/A	N/A	N/A	N/A
Total	0.22		0.17		N/A		N/A	
**Seq. Coverage**	3.09×	0.31×	5.2×	1.1×	7.0×	N/A	7.0×	N/A
Total	3.4×		6.3×		7.0×		7.0×	
**Clone Coverage**	6.02×	1.00×	10.3×	3.6×	N/A	N/A	N/A	N/A
Total	7.02×		13.9×		N/A		N/A	

The assembled contigs and scaffolds of the Btau_3.1 assembly were placed on the chromosomes using a version of the Integrated Bovine Map that represents merged data from several independent maps[11]. Btau_4.0 is the latest assembly. This assembly added relatively little new sequence data, and thus contigs and scaffolds were not significantly changed, but used the ILTX[12] and BAC finger-print contig [11] maps and split scaffolds based on consistent bovine and sheep BES data [13] to place contigs and scaffolds in the genome, instead of the Integrated Bovine Map, resulting in more accurate chromosome structures.

Overall, 90% of the total genome was placed on chromosomes in the Btau_4.0 assembly (Table 3). This assembly was tested against available bovine sequence data sets (Tables 4 and Additional file 1). Of the 1.04 million EST sequences, 95.0% were contained in the assembled contigs. Assuming the ESTs are uniformly distributed throughout the genome, the estimated genome size is 2.87 Gb (2.73 Gb/0.95). The quality of the assembly was also tested by alignment to 73 finished BACs. The genomic coverage in these BACs was high, between 92.5% and 100.0% (average of 98.5%) of the BAC sequence in the assembly. The assembled contigs and scaffolds were aligned linearly to the finished BACs, suggesting that misassemblies are rare.

**Table 3:** Scaffold Placement Statistics for Btau_4.0

Scaffolds/Contigs	Number	N50 (kbp)	Total (Gbp)	Percent*
Anchored & Oriented	2,194	2,187	2.54	89
Anchored Not Oriented	137	500	0.04	1
Unanchored Scaffolds	11,830	94	0.28	10

*Percentage of genome sequence in each category. Scaffolds includes single contig scaffolds as well as multiple contig scaffolds.

**Table 4:** Assembly Completeness

Percentage Matched	Btau 1.0	Btau 2.0	Btau 3.1	Btau 4.0
Finished BACs	18	N/A	73	73
Contigs	94.57	N/A	98.50	98.50
Markers	N/A	10,387	21,971	21,971
Scaffolds	N/A	95.70	98.61	98.61
Unigene sets or ESTs	23,924	23,924	1,040,000	1,040,000
Scaffolds	83.82	92.40	95.00	95.00
BES	N/A	321,287	N/A	N/A
Scaffolds	N/A	95.20	N/A	N/A

The table gives the numbers of BACS, markers, ESTs or BES used for comparison, and the percentage of Contigs or Scaffolds that matched each set. Additional file 2 gives the same comparisons for the unassembled reads as well as the assembled sequences.

Two groups have used SNP linkage data to order scaffolds on particular chromosomes. One group used SNP linkage data to order scaffolds on Chr6 [14] and another placed scaffolds on Chr19 and Chr29 [15]. Their studies provided additional evidence for scaffold placements and independent measurements for the quality of the assembly. Scaffolds in Btau_4.0 have an order entirely consistent with the evidence from these three chromosomes, while both Btau_3.1 and the composite map[11] show misplaced scaffolds (see the summary in Table 5, and details in Additional file 2).

**Table 5:** Comparison to Independent Chromosome Maps

		Misplaced in Scaffolds		
		
	Total Shared	Btau_4.0	Btau_3.1	Integrated Bovine Map[11]
	
Chr6	61	0	15	7
Chr19	45	0	6	9
Chr29	28	0	7	7

Further assessment of the Btau_4.0 assembly was performed by comparing dense SNP linkage maps constructed from genotyping 17,482 SNPs in 2,637 bulls belonging to 108 half-sib families with the physical positioning of the SNPs on all autosomal chromosomes. The analysis revealed that 134 SNPs were incorrectly positioned within assembly. This relatively small number (<0.8%) indicates the high degree of precision in the Btau_4.0 assembly. These misplaced SNPs were relocated in the linkage map to a position corresponding to the most closely linked, correctly assigned SNP. Additionally, 568 SNPs from 321 unplaced scaffolds were mapped to linkage groups.

## Discussion

The genome assembly version Btau_4.0 is available in GenBank under accession number AAFC0000000.3. In addition, the individual components of the genome assembly (the sequence contigs and corresponding quality files) and the file with the component assembly instructions (the .agp format file) are available from the BCM-HGSC ftp site[16] which is a link from the bovine page on the BCM-HGSC web site.) [17] Since the process of genome assembly involves decisions about which sequences to include and which sequences to exclude, there are sequences from this project that were omitted from the final assembly. Some of the omitted sequences are highly repetitive sequence reads, others may have enough sequencing errors that they did not match the assembled sequences, others are assembled sequence contigs that appear to be duplicates of sequences in the assembly (perhaps from the second haplotype). These excluded sequence are also available from the BCM-HGSC ftp site.

The majority of the sequence in the project is from the female animal, the genome sequence is described for the 29 autosomes and the X chromosome. However, as the BAC library was prepared from a male animal, and the BAC fingerprint contigs were built from random clones from that library, both the X and Y chromosomes are represented in the BAC fingerprint contigs. Representative BACs in all of the BAC fingerprint contigs were sequenced to low coverage, including Y chromosome BACs. Since the clone coverage on the sex chromosomes in the BAC library is half that of the autosomes, there will be less depth of clone coverage on the sex chromosomes and this may result in more gaps in the coverage of the sex chromosomes by BAC clones. The WGS sequence was from the female animal, so there is not additional WGS sequence to assemble with the low coverage BAC skim sequences for the Y chromosome, unless it is pseudoautosomal sequence from the X chromosome or autosomal sequence that is similar to the Y sequence. Since the BAC fingerprint contigs were used to build the combined BAC+WGS assemblies, there are genome sequence scaffolds from both sex chromosomes as well as the autosomes. The Y chromosome scaffolds are unlabeled in the unplaced chromosome.

The use of linkage data to identify incorrectly positioned SNPs has been extended to also reveal the identity of the most closely linked, and correctly positioned SNPs. In addition, the identification of 568 SNPs that map to linkage groups but are found within 321 unplaced scaffolds can be used to suggest a position for these scaffolds within the assembly. Taken together, data associating misplaced SNPs and unplaced scaffolds with correctly positioned markers could be used to highlight regions that could benefit from map assisted assembly improvements. However, the moderate number of individuals being genotyped (2,637 bulls) limits the mapping resolution. So while this analysis is effective at resolving large distance misplacements, additional genotyping of families would be required to reveal more local rearrangements.

## Conclusion

The bovine genome assembly reported here was used for the analysis of the bovine genome sequence that is being published. Most of those analyses used the gene annotation from the Btau_3.1 assembly. Some of the analyses used the Btau_4.0 assembly.

## Methods

### Description of the WGS only assembly

Two assembly versions were prepared using only whole genome shotgun (WGS) reads from small insert clones and BAC end sequences (BES). The DNA for the small insert WGS libraries was from white blood cells from the Hereford cow L1 Dominette 01449, American Hereford Association registration number 42190680 (provided by Dr. Timothy Smith, U.S. Meat Animal Research Center, Clay Center, NE). The inbreeding coefficient was 31%. These WGS assemblies did not include sample sequence from the BAC clones. Btau_1.0 (September 2004) was produced with about 3× WGS coverage. Btau_2.0 (June 2005) was produced with about 6.2× WGS coverage.

The Btau_2.0 release was produced by assembling WGS reads with the Atlas genome assembly system[10]. Several WGS libraries, with inserts of 2–4 kb, and 4–6 kb, were used to produce the data. About 23 million reads were assembled, representing about 17.7 Gb of sequence and about 6.2× coverage of the (clonable) bovine genome (see Tables 1 and 2). BES were used for scaffolding.

The products of the Atlas assembler are a set of contigs (contiguous blocks of sequence) and scaffolds. Scaffolds include sequence contigs that can be ordered and oriented with respect to each other as well as isolated contigs that could not be linked (single contig scaffolds or singletons). Reads which clustered into groups of 3 or fewer were not assembled. The N50 size of the contigs in the Btau_2.0 assembly is 18.9 kb and the N50 of the scaffolds is 434.7 kb (Table 6). The N50 size is the length such that 50% of the assembled genome lies in blocks of the N50 size or longer. The total length of all contigs is 2.62 Gb. When the gaps between contigs in scaffolds are included, the total span of the assembly is 3.1 Gb (some scaffolds with large gaps may artificially increased the assembly size).

**Table 6:** Assembly contig and scaffold statistics

	**Btau 1.0**	**Btau 2.0**	**Btau 3.1**	**Btau 4.0**
**Contigs**				
**Number**	795,212	321,107	131,620	131,620
**N50(kb)**	4.20	18.90	48.70	48.70
**Bases+Gaps(Gb)**	2.26	2.62	2.73	2.73
**Bases(Gb)**	2.26	2.62	2.73	2.73
**Percentage**		85	95.1	95
**Anchored and Oriented Scaffolds**				
**Number**	0	2215	2,055	2,194
**N50(kb)**		712	1,393	2,187
**Bases+Gaps(Gb)**		1.07	2.08	2.54
**Bases(Gb)**		0.89	1.99	2.43
**Percentage**	0	34.5	72.7	89
**Anchored and Unoriented Scaffolds**				
**Number**	0	2194	998	137
**N50(kb)**		535	547	500
**Bases+Gaps(Gb)**		0.63	0.32	0.04
**Bases(Gb)**		0.51	0.3	0.04
**Percentage**	0	20.3	11	1
**Unanchored Scaffolds**				
**Number**	449,727	98,058	13,045	11,830
**N50(kb)**	13.5	189	166	94
**Bases+Gaps(Gb)**	2.34	1.4	0.47	0.28
**Bases(Gb)**	2.26	1.2	0.44	0.26
**Percentage**	100	45.2	16.3	10
**Total Scaffolds**				
**Number**	449,727	102,467	16,098	14,161
**N50(kb)**	13.5	434	997	1,922
**Bases+Gaps(Gb)**	2.34	3.1	2.87	2.87
**Bases(Gb)**	2.26	2.62	2.73	2.73
**Percentage**	100	100	100	100

The Btau_2.0 assembly was tested against available bovine sequence data sets (EST sequences, Unigene clusters, BES and finished BAC sequences) for extent of coverage (completeness) (see Table 4 and Additional file 1). When all sequences (assembled contigs and unassembled reads) were tested, over 95% of the sequences in these data sets were found to be represented, indicating that the shotgun libraries used to sequence the genome were comprehensive.

### Description of the BAC based assemblies

Btau_3.1 (August 2006) was produced with a combination of WGS and BAC sequence by the Atlas genome assembly system[10]. The source of the BAC library DNA was Hereford bull L1 Domino 99375, registration number 41170496 (father of L1 Dominette 01449; Dr. Michael MacNeil's laboratory, USDA-ARS, Miles City, MT provided the blood). The assembly process consisted of multiple phases (see Figure 1). BACs were sequenced as either individual clone libraries or as pools of arrayed clones (see read statistics in Table 1). BAC reads from individual libraries or from deconvoluted pools were assembled as individual BACs. 19,667 BAC projects (12,549 individual sequenced clones and 7,118 clones from BAC pools) were sequenced and assembled.

Individual BAC sequences were assembled with Phrap[18,19], first with just the BAC generated sequences, then in combination with the WGS reads that overlap the BAC as an enriched BAC (eBAC). Three assembly methods were applied to each individual eBAC using the BAC reads and the WGS reads that overlapped with the BAC reads: 1) PHRAP: eBAC assemblies were produced by Phrap[18] using either raw or trimmed reads. The better assembly result from the two read sets was determined based on contig and scaffold size statistics. 2) SPLIT: The positions of potential misjoins in the contigs generated from method (1) were detected when a region in a contig had a lack of clone coverage and contained conflicting clone links with the other contigs. The reads in this region were removed and Phrap[18] assembly was performed again to split the original contig. These contigs were named e.g. Contig22.CH240-403F14.split. 3) WGS: Each individual eBAC was treated as a mini-genome and the standard ATLAS-WGS assembly procedure was applied, including detecting overlaps among the reads, filtering conflicting overlaps based on overlap patterns, clustering reads into bins based on their overlaps and PHRAP assembly in each bin. These contigs were named e.g. Contig17.CH240-105B18.wgs. These three assembly methods were implemented as new components that have been added to the Atlas assembly system.

For any BAC, the assembly using one of the above three methods was selected (based on the sequence alignment of this BAC against the BACs that overlapped with it) and used in the next step of BAC merging. The BAC merging used the eBAC scaffold merger developed for sea urchin rather than the rolling phrap method used for the rat. Briefly, the combined read set assemblies for each BAC were refined by contig merging and scaffolding based on clone-end mate pair constraints. Sets of overlapping BAC clones were identified and merged based on shared WGS reads and sequence overlaps of individual BAC assemblies. The merged BAC assemblies were further scaffolded using information from mate pairs, BAC clone vector locations, and BAC assembly sequences.

### Description of the merging process combining BAC based and WGS only assemblies

Contigs from the Btau_2.0 WGS assembly were used to fill in the gaps in the BAC-based assembly (e.g. those due to gaps in the BAC tiling path). In the combined assembly, Btau_3.1, the N50 size of the contigs is 48.7 kb and the N50 of the scaffolds is 997.5 kb (Table 6). The total length of all contigs is 2.73 Gb. When the gaps between contigs in scaffolds are included, the total span of the assembly is 2.87 Gb (some scaffolds with large gaps may artificially increased the assembly size). The assembly includes a total of 26,052,388 reads, which yields a ~7.0× sequence coverage (using the average trimmed read length as 730 bp and the assembly size as 2.73 Gb). The Btau_3.1 assembly was tested against available bovine sequence data sets for completeness (Table 4 and Additional file 1).

### Description of mapping and placement for Btau_3.1

The assembled contigs and scaffolds of the Btau_3.1 assembly were placed on the chromosomes using an early version of the Integrated Bovine Map[11] that represents merged data from several independent maps. A total of 21,971 bovine markers were compared to the Btau_v3.1 scaffolds using MegaBLASTN[20] (see Table 7). The vast majority of the markers (21,666) have matches to the assembly (Table 7). The MegaBLAST results were first filtered by requiring matches to at least 40% of the marker length at at least 90% match identity. Repeat filtering removed markers with match scores of the top hits that were within 50 points of each other.

**Table 7:** Marker Statistics for Btau_3.1

Marker	Number in Btau_3.1
Total assessed	21,971
With matches	21,666
Without matches	305
Low identity matches	1,670
Repeat matches	1,606
Removed due to conflicts	595
Total used in final map	17,795

After filtering, scaffolds with markers were anchored onto the chromosomes according to the marker orders provided in the integrated map. In the cases where a scaffold had markers from different chromosomes, the scaffold was checked for dog and human synteny. If the synteny information confirmed that the scaffold should be on different chromosomes, the scaffold was split. Otherwise, the minor group(s) of the markers were ignored. In the cases where a scaffold had markers from a single chromosome but the markers were far apart, the scaffold was anchored by the major group of the markers. In the cases where the markers were on a single chromosome but the integrated map marker order was not consistent with the mapping on the genome scaffold assemblies, the marker order was rearranged according to the scaffold sequences. The scaffold orientation on the chromosome was determined by the order of the markers. When it was impossible to determine the orientation (e.g. a scaffold with a single marker), the scaffolds were labeled as unoriented.

### Description of refined mapping and placement for Btau_4.0

Btau_4.0 is the latest (as of Oct. 4, 2007) assembly of the genome of Bos taurus, Hereford breed. This assembly added relatively little new sequence data, and thus contigs and scaffolds are not significantly changed, but used different map information than was used for the Btau_3.1 assembly to place the contigs and scaffolds in the genome, resulting in more accurate chromosome structures. The mapping procedure is described below.

BES reads from both Hereford (189,587) and Non-Hereford (131,700) breeds were aligned to the scaffolds using BLASTN and clone links were used to generate a set of larger scaffolds. Scaffolds that had potential misassemblies were split based on Bovine and Sheep BES links[13] when the bovine and sheep BES consistently indicated that the parts of the scaffold mapped to different regions. After splitting, the scaffolds were mapped to the chromosomes based on the ILTX marker map[12]. The positions of the markers on the scaffolds were determined by BLASTN alignment.

The order of the scaffolds on the chromosomes was refined based on the information from three sources: the fingerprint contig map (FPC)[21], human and dog synteny, and links by sheep BAC clones[13]. When any three adjacent scaffolds had order information from at least two of the three sources and the order was consistent among these sources but in conflict with the ILTX map[12], the order of the scaffolds was modified from the ILTX map order[12]. The scaffolds that were not oriented by the ILTX map[12] were oriented using the FPC information when such information was available.

Additional scaffolds were placed if two adjacent scaffolds from above were present in the FPC map[21] and there were additional scaffolds in the FPC map between them. These additional scaffolds from FPC were filled in on the chromosomes.

The remaining un-oriented scaffolds were further oriented based on Human Synteny. This step oriented ~9% of the scaffolds. Additional scaffolds were mapped to the chromosomes based on the Bovine and Sheep BES links with the supporting evidence from the FPC[21] and SNP maps. Finally, when various sources suggested different locations of scaffolds, the ambiguity was resolved where possible by checking the synteny and the individual eBAC assemblies. Overall, 90% of the total genome was placed on chromosomes (Table 3 and Additional file 1).

### Evaluation of the Btau_4.0 assembly

The Btau_4.0 assembly was tested against available bovine sequence data sets (Table 4 and Additional file 1). Of the 1.04 million EST sequences 95.0% were contained in the assembled contigs. Assuming the ESTs are uniformly distributed throughout the genome, the estimated genome size is 2.73 Gb/95% = 2.87 Gb. The quality of the assembly was also tested by alignment to the 73 finished BACs. The genomic coverage in the BACs was high, between 92.5% and 100.0% (average of 98.5%) of the BAC sequence in the assembly. The assembled contigs and scaffolds were aligned linearly to the finished BACs, suggesting that misassemblies are rare.

The accuracy of marker positions in the genome is reflected by the order of scaffolds on the chromosomes as scaffolds were placed on chromosomes based on their alignments to markers. Two groups have used their marker sets to order scaffolds in high confidence on particular chromosomes. SNP linkage data discussed for the whole genome in more detail below was initially available for Chr6[14] and Steve Moore's group placed scaffolds on Chr19 and Chr29[15]. These studies thus provided additional evidence for scaffold placements and independent measurements for the quality of the assembly.

For these three chromosomes, we compared the order of scaffolds with the independent mapping evidence for three datasets: Btau_3.1 which used an early version of the Integrated Bovine Map[11], Btau_4.0, and the scaffold order using the published version of the Integrated Bovine Map[11]. The comparison showed consistency between the evidence and Btau_4.0, i.e. all the scaffolds in Btau_4.0 were in increasing order. In contrast, conflicts occurred when comparing the evidence with Btau_3.1. Most of the inconsistencies happened between neighboring scaffolds, suggesting that errors in the order of Btau_3.1 markers were primarily local errors. Chr6 clearly had many more errors in Btau_3.1 than Chr19 and Chr29. The published version of the Integrated Bovine Map showed fewer conflicts with the evidence overall (e.g. Chr6) than the version of the Integrated Bovine Map used in Btau_3.1 although the differences did not necessarily solve the conflicts and in some cases even generated new inconsistencies (e.g. Chr19). Table 5 is the summary of the number of misplaced scaffolds in three data sets (Btau_4.0; Btau_3.1; and the Integrated Bovine Map[11]) for three chromosomes when compared with the independent mapping evidence. More complete data is given in Additional file 2.

### Quality assessment of the assembly by linkage analysis

Norwegian Red cattle (2,637) within a paternal halfsib pedigree structure were genotyped using the Affymetrix 25 K MIP array. Quality checking of the data revealed that almost 30% of SNP assays were generating unreliable or uninformative genotypes. Consequently, allele calls from only 17,482 SNPs were included in linkage analysis using CRIMAP 2.4[22]. The initial SNP order employed in the linkage analysis was based upon the Btau_4.0 assembly. The *chrompic *function of CRIMAP was used to detect possible genotyping errors and SNP misplacements as indicated by double recombinants within an individual's chromosome. SNPs identified as being suspicious (i.e. double recombinants) were removed from the linkage map and scanned against all remaining SNPs using CRIMAP's *twopoint *option. This analysis identified 134 SNPs (less than 0.8%) that mapped more strongly to positions in the genome other than those originally suggested by the Btau_4.0 assembly.

To highlight instances where several SNPs within a relatively small physical region were being relocated, the exact SNP positions were rounded up to the nearest whole Mb value; in so doing SNPs within a 1 Mb region of sequence were effectively binned together. Before repositioning, markers were clustered in one of six 2-SNP bins, two 3-SNP bins, or two 5-SNP bins, with the remaining 106 SNPs separated from each other by distances greater than 1 Mb (see Additional file 3 part A). After repositioning, 98 SNPs remained isolated, but the number of 2-SNP bins had increased to 15, and there was one bin containing 6 SNPs (see Additional file 3 part B). Details of the repositioning are presented in Additional file 4.

In addition to repositioning of SNPs, the construction of linkage groups enabled placement of SNPs with previously unknown positions. SNP markers (568) distributed across 321 scaffolds were placed throughout the autosomes as shown in Additional file 5. As in the repositioning analysis described above, this placement analysis clustered SNPs into 1 Mb bins to highlight co-placements. Three-hundred SNPs were found to cluster into bins, with twenty 2-SNP bins, five 3-SNP bins, four 5-SNP bins, five 6-SNP bins, one 7-SNP bin, two 8-SNP bins, and one each of 9-SNP and 10-SNP bins. See Additional file 6 for the placement details.

Repositioning or placement of binned SNPs (i.e. >2 SNPs within a 1 Mb region) can indicate either translocation of large blocks, or amalgamation of small fragments. Movement of larger bins (especially those found during placement analysis) highlights those regions that were lacking data and may indicate that these regions contain difficult to assemble sequence motifs such as repeats.

## Abbreviations

**BAC**: bacterial artificial chromosome; **BES**: BAC end sequences; **BCM-HGSC**: Human Genome Sequencing Center, Baylor College of Medicine; **eBAC**: enriched BAC (assembled with BAC sequence and overlapping WGS sequence); **EST**: expressed sequence tag; **FPC**: fingerprint contig; **SNP**: single nucleotide polymorphism; **WGS**: whole genome shotgun.

## Authors' contributions

YL produced the final assembly, developed methods for using different BAC assembly methods and combining the BAC and WGS assemblies. XQ produced the whole genome shotgun assemblies and performed mapping of the markers to these assemblies. XHS performed the synteny mapping to other mammalian genomes. HJ performed the BAC assemblies of pooled BACs and eBACs. YS modified methods he developed for sea urchin dealing with pooled BACs and merging BACs so that they could be used in the bovine project. KJD modified his code for merging BACs, advised on deconvolution of pooled BACs. SL, MS and MPK contributed mapping information and examined linkage data for all autosomes to quality check the assembly. YR provided read wrangling support by collecting sequence data and building the reads database prior to assembly. LZ evaluated paired-end data to quality check the assemblies. ES managed the BAC and pooled BAC processing and consulted on the use of that data. PH adjusted the software for the BAC-fishing assemblies and advised on the deconvolution of pooled BACs. KCW directed the genome assembly group and provided guidance and coordination, contributed to writing the manuscript. GW, co-director of the HGSC during this project, provided direction and coordination with the bovine community. RAG director of the HGSC, secured funding and provided project coordination and direction.

## Supplementary Material

Additional file 1 **Completeness of assembly compared to unassembled reads**. Table provides completeness statistics for 4 assemblies compared to finished BACs, markers, ESTs, and BAC end sequences. Click here for file

Additional file 2 **Detailed comparisons of Independent Maps**. Table for comparison of independent maps of chromosomes 6, 19 and 29. Each column gives the order of the scaffolds in the map. Column 1 is the scaffold name, column 2 is the order in the chromosome map used as the gold standard evidence, column 3 is the order in the Btau_4.0 assembly, column 4 is the order in the Integrated Bovine Map[11], column 5 is the order in the Btau_3.1 assembly. Click here for file

Additional file 3 **SNP distribution before and after repositioning**. Figure shows the locations of the small fraction of SNPs (135 SNPs, or 0.8%) whose LOD scores were found to improve with repositioning are shown. The SNPs were grouped into local 1 Mb sized bins. Bins with more than one SNP are identified with different indicators on the graphs. (A) The locations of the SNPs **before **repositioning. (B) The locations the SNPs **after **repositioning. Click here for file

Additional file 4 **SNPs with linkage position different from Btau_4.0 assembly postion**. Table provides list of SNPs with linkage positions that disagree with Btau_4.0 assembly, also provides the identity and position of the most closely linked SNP. Columns include SNP name, chromosome, position in Btau_4.0, and best two-point hit, with the chromosome and position for that linked SNP. Click here for file

Additional file 5 **Additional SNP placement by linkage analysis**. The locations of 568 SNPs with previously unassigned position in Btau_4.0 whose location was determined by identifying the pairwise comparison between the unknown SNP and all the mapped SNPs that produced the highest LOD score using the *twopoint *option of CRIMAP. Click here for file

Additional file 6 **Placement of unplaced scaffolds using linkage information**. Table provides placement information for unplaced scaffolds based on linked markers. Columns include SNP, unplaced Contig, location in unplaced contig, chromosome placement, linked SNP, location in chromosome. Click here for file
