# Supplementary material for: Bos taurus genome assembly
Source: BMC Genomics. 2009 Apr 24;10:180. doi: 10.1186/1471-2164-10-180 (PMC2686734; doi:10.1186/1471-2164-10-180)
Supplement: Additional file 1 — Completeness of assembly compared to unassembled reads. Table provides completeness statistics for 4 assemblies compared to finished BACs, markers, ESTs, and BAC end sequences. [file 1471-2164-10-180-S1.doc]

**Additional file 1 - Completeness of assembly compared to unassembled reads**

| **Completeness** | **Percentage** | **Btau 1.0** | **Btau 2.0** | **Btau 3.1** | **Btau 4.0** |
| --- | --- | --- | --- | --- | --- |
| **Finished Bacs** |  | **18** |  | **73** | **73** |
| Matched Contigs | Assembly | 94.57 % | N/A | 98.50 % | 98.50 % |
| **Markers** |  |  | **10,387** | **21,971** | **21,971** |
| Matched Scaffolds | Assembly | N/A | 95.70 % | 98.61 % | 98.61 % |
| Matched Scaffolds | Unassembled reads | N/A | 18.80 % | N/A | N/A |
| Matched Scaffolds | Assembly + Unassembled reads | N/A | 95.80 % | N/A | N/A |
| **Unigene sets or ESTs** |  | **23,924** | **23,924** | **1,040,000** | **1,040,000** |
| Matched Scaffolds | Assembly | 83.82 % | 92.40 % | 95.00 % | 95.00 % |
| Matched Scaffolds | Unassembled reads | 11.83 % | 5.50 % | N/A | N/A |
| Matched Scaffolds | Assembly + Unassembled reads | 85.81 % | 92.50 % | N/A | N/A |
| **BES** |  |  | **321,287** |  |  |
| Matched Scaffolds | Assembly | N/A | 95.20 % | N/A | N/A |
| Matched Scaffolds | Unassembled reads | N/A | 5.00 % | N/A | N/A |
| Matched Scaffolds | Assembly + Unassembled reads | N/A | 95.50 % | N/A | N/A |
