# Supplementary material for: Bos taurus genome assembly
Source: BMC Genomics. 2009 Apr 24;10:180. doi: 10.1186/1471-2164-10-180 (PMC2686734; doi:10.1186/1471-2164-10-180)
Supplement: Additional file 2 — Detailed comparisons of Independent Maps. Table for comparison of independent maps of chromosomes 6, 19 and 29. Each column gives the order of the scaffolds in the map. Column 1 is the scaffold name, column 2 is the order in the chromosome map used as the gold standard evidence, column 3 is the order in the Btau_4.0 assembly, column 4 is the order in the Integrated Bovine Map[11], column 5 is the order in the Btau_3.1 assembly. [file 1471-2164-10-180-S2.doc]

**Additional file 2. Detailed comparisons of Independent Maps**

Each column gives the order of the scaffolds in the map. Column 1 is the scaffold name, column 2 is the order in the chromosome map used as the gold standard evidence, column 3 is the order in the Btau_4.0 assembly, column 4 is the order in the Integrated Bovine Map[11], column 5 is the order in the Btau_3.1 assembly.

1) Chr6

Scaffold Name Evidence Btau_4.0 Int_Bov_Map Btau_3.1

gnl|Btaurus3.1|Chr6.2 1 5 5 2

gnl|Btaurus3.1|Chr6.1 2 7 8 1

gnl|Btaurus3.1|Chr6.7 3 9 9 7

gnl|Btaurus3.1|Chr6.6 4 10 11 6

gnl|Btaurus3.1|Chr6.14 5 12 12 14

gnl|Btaurus3.1|Chr6.13 6 13 14 13

gnl|Btaurus3.1|Chr6.11 7 15 16 11

gnl|Btaurus3.1|Chr6.23 8 18 18 23

gnl|Btaurus3.1|Chr6.17 9 19 21 17

gnl|Btaurus3.1|Chr6.24 10 21 19 24

gnl|Btaurus3.1|Chr6.18 11 22 22 18

gnl|Btaurus3.1|Chr6.21 12 23 24 21

gnl|Btaurus3.1|Chr6.19 13 25 25 19

gnl|Btaurus3.1|Chr6.26 14 26 28 26

gnl|Btaurus3.1|Chr6.28 15 28 31 28

gnl|Btaurus3.1|Chr6.29 17 29 32 29

gnl|Btaurus3.1|Chr6.30 18 30 33 30

gnl|Btaurus3.1|Chr6.34 19 32 36 34

gnl|Btaurus3.1|Chr6.31 20 33 29 31

gnl|Btaurus3.1|Chr6.35 21 34 37 35

gnl|Btaurus3.1|Chr6.36 22 35 34 36

gnl|Btaurus3.1|Chr6.39 23 38 40 39

gnl|Btaurus3.1|Chr6.40 24 39 41 40

gnl|Btaurus3.1|Chr6.41 25 40 42 41

gnl|Btaurus3.1|Chr6.43 26 41 43 43

gnl|Btaurus3.1|Chr6.42 27 42 44 42

gnl|Btaurus3.1|Chr6.46 28 45 45 46

gnl|Btaurus3.1|Chr6.47 29 46 48 47

gnl|Btaurus3.1|Chr6.48 30 48 50 48

gnl|Btaurus3.1|Chr6.50 31 50 51 50

gnl|Btaurus3.1|Chr6.52 32 51 53 52

gnl|Btaurus3.1|Chr6.55 33 54 56 55

gnl|Btaurus3.1|Chr6.57 34 56 57 57

gnl|Btaurus3.1|Chr6.59 35 59 61 59

gnl|Btaurus3.1|Chr6.60 36 61 63 60

gnl|Btaurus3.1|Chr6.61 37 62 64 61

gnl|Btaurus3.1|Chr6.63 38 64 65 63

gnl|Btaurus3.1|Chr6.66 39 65 67 66

gnl|Btaurus3.1|Chr6.67 40 67 68 67

gnl|Btaurus3.1|Chr6.69 41 68 69 69

gnl|Btaurus3.1|Chr6.70 42 69 71 70

gnl|Btaurus3.1|Chr6.72 43 73 75 72

gnl|Btaurus3.1|Chr6.80 44 77 79 80

gnl|Btaurus3.1|Chr6.81 45 79 81 81

gnl|Btaurus3.1|Chr6.83 46 81 82 83

gnl|Btaurus3.1|Chr6.84 47 83 83 84

gnl|Btaurus3.1|Chr6.76 48 85 87 76

gnl|Btaurus3.1|Chr6.89 49 88 89 89

gnl|Btaurus3.1|Chr6.88 50 89 90 88

gnl|Btaurus3.1|Chr6.97 51 90 91 97

gnl|Btaurus3.1|Chr6.91 52 91 92 91

gnl|Btaurus3.1|Chr6.90 53 92 93 90

gnl|Btaurus3.1|Chr6.96 54 96 99 96

gnl|Btaurus3.1|Chr6.101 55 98 97 101

gnl|Btaurus3.1|Chr6.95 56 101 102 95

gnl|Btaurus3.1|Chr6.99 57 102 103 99

gnl|Btaurus3.1|Chr6.103 58 104 104 103

gnl|Btaurus3.1|Chr6.108 60 111 115 108

gnl|Btaurus3.1|Chr6.111 61 112 112 111

gnl|Btaurus3.1|Chr6.127 62 113 116 127

gnl|Btaurus3.1|Chr6.112 63 114 117 112

2) Chr19

Scaffold Name Evidence Btau_4.0 Int_Bov_Map Btau_3.1

gnl|Btaurus3.1|Chr19.1 1 2 4 1

gnl|Btaurus3.1|Chr19.4 2 5 6 4

gnl|Btaurus3.1|Chr19.5 3 6 1 5

gnl|Btaurus3.1|Chr19.6 4 7 9 6

gnl|Btaurus3.1|Chr19.11 5 9 10 11

gnl|Btaurus3.1|Chr19.9 6 11 8 9

gnl|Btaurus3.1|Chr19.13 7 12 12 13

gnl|Btaurus3.1|Chr19.12 8 13 13 12

gnl|Btaurus3.1|Chr19.14 9 15 14 14

gnl|Btaurus3.1|Chr19.16 10 16 17 16

gnl|Btaurus3.1|Chr19.17 11 18 16 17

gnl|Btaurus3.1|Chr19.19 13 19 18 19

gnl|Btaurus3.1|Chr19.21 14 20 21 21

gnl|Btaurus3.1|Chr19.23 16 21 23 23

gnl|Btaurus3.1|Chr19.25 17 23 24 25

gnl|Btaurus3.1|Chr19.27 19 24 27 27

gnl|Btaurus3.1|Chr19.28 20 25 29 28

gnl|Btaurus3.1|Chr19.30 21 27 30 30

gnl|Btaurus3.1|Chr19.32 23 30 33 32

gnl|Btaurus3.1|Chr19.33 24 31 35 33

gnl|Btaurus3.1|Chr19.36 25 32 34 36

gnl|Btaurus3.1|Chr19.35 26 33 36 35

gnl|Btaurus3.1|Chr19.37 27 34 37 37

gnl|Btaurus3.1|Chr19.38 28 35 38 38

gnl|Btaurus3.1|Chr19.39 29 36 39 39

gnl|Btaurus3.1|Chr19.43 31 40 44 43

gnl|Btaurus3.1|Chr19.40 32 41 40 40

gnl|Btaurus3.1|Chr19.47 33 42 48 47

gnl|Btaurus3.1|Chr19.50 34 43 47 50

gnl|Btaurus3.1|Chr19.48 35 44 49 48

gnl|Btaurus3.1|Chr19.49 36 45 42 49

gnl|Btaurus3.1|Chr19.51 37 46 51 51

gnl|Btaurus3.1|Chr19.52 38 47 50 52

gnl|Btaurus3.1|Chr19.54 39 49 53 54

gnl|Btaurus3.1|Chr19.58 40 52 59 58

gnl|Btaurus3.1|Chr19.55 41 53 54 55

gnl|Btaurus3.1|Chr19.56 43 55 56 56

gnl|Btaurus3.1|Chr19.63 44 56 62 63

gnl|Btaurus3.1|Chr19.65 45 58 64 65

gnl|Btaurus3.1|Chr19.66 46 59 65 66

gnl|Btaurus3.1|Chr19.68 47 60 67 68

gnl|Btaurus3.1|Chr19.69 48 61 68 69

gnl|Btaurus3.1|Chr19.71 49 62 70 71

gnl|Btaurus3.1|Chr19.72 51 63 71 72

gnl|Btaurus3.1|Chr19.73 52 64 69 73

3) Chr29

Scaffold Name Evidence Btau_4.0 Int_Bov_Map Btau_3.1

gnl|Btaurus3.1|Chr29.10 1 10 10 10

gnl|Btaurus3.1|Chr29.14 2 14 14 14

gnl|Btaurus3.1|Chr29.27 3 15 27 27

gnl|Btaurus3.1|Chr29.17 4 16 17 17

gnl|Btaurus3.1|Chr29.16 5 17 16 16

gnl|Btaurus3.1|Chr29.18 6 18 18 18

gnl|Btaurus3.1|Chr29.20 7 19 20 20

gnl|Btaurus3.1|Chr29.22 8 20 22 22

gnl|Btaurus3.1|Chr29.21 9 21 21 21

gnl|Btaurus3.1|Chr29.23 10 22 23 23

gnl|Btaurus3.1|Chr29.25 12 23 25 25

gnl|Btaurus3.1|Chr29.36 13 33 36 36

gnl|Btaurus3.1|Chr29.37 14 34 37 37

gnl|Btaurus3.1|Chr29.41 15 35 41 41

gnl|Btaurus3.1|Chr29.40 16 37 40 40

gnl|Btaurus3.1|Chr29.44 17 40 44 44

gnl|Btaurus3.1|Chr29.45 18 41 45 45

gnl|Btaurus3.1|Chr29.47 19 42 47 47

gnl|Btaurus3.1|Chr29.50 20 45 50 50

gnl|Btaurus3.1|Chr29.49 21 46 49 49

gnl|Btaurus3.1|Chr29.53 22 47 52 53

gnl|Btaurus3.1|Chr29.48 23 49 48 48

gnl|Btaurus3.1|Chr29.59 25 57 59 59

gnl|Btaurus3.1|Chr29.62 26 58 62 62

gnl|Btaurus3.1|Chr29.64 27 59 64 64

gnl|Btaurus3.1|Chr29.63 28 60 63 63

gnl|Btaurus3.1|Chr29.65 29 61 65 65

gnl|Btaurus3.1|Chr29.66 30 62 66 66
