# Supplementary material for: Bos taurus genome assembly
Source: BMC Genomics. 2009 Apr 24;10:180. doi: 10.1186/1471-2164-10-180 (PMC2686734; doi:10.1186/1471-2164-10-180)
Supplement: Additional file 3 — SNP distribution before and after repositioning. Figure shows the locations of the small fraction of SNPs (135 SNPs, or 0.8%) whose LOD scores were found to improve with repositioning are shown. The SNPs were grouped into local 1 Mb sized bins. Bins with more than one SNP are identified with different indicators on the graphs. (A) The locations of the SNPs before repositioning. (B) The locations the SNPs after repositioning. [file 1471-2164-10-180-S3.doc]

**Additional file 3. SNP distribution before and after repositioning**

The locations of the small fraction of SNPs (135 SNPs, or 0.8%) whose LOD scores were found to improve with repositioning are shown. The SNPs were grouped into local 1Mb sized bins. Bins with more than one SNP are identified with different indicators on the graphs. (A) The locations of the SNPs **before** repositioning. (B) The locations the SNPs **after** repositioning. **
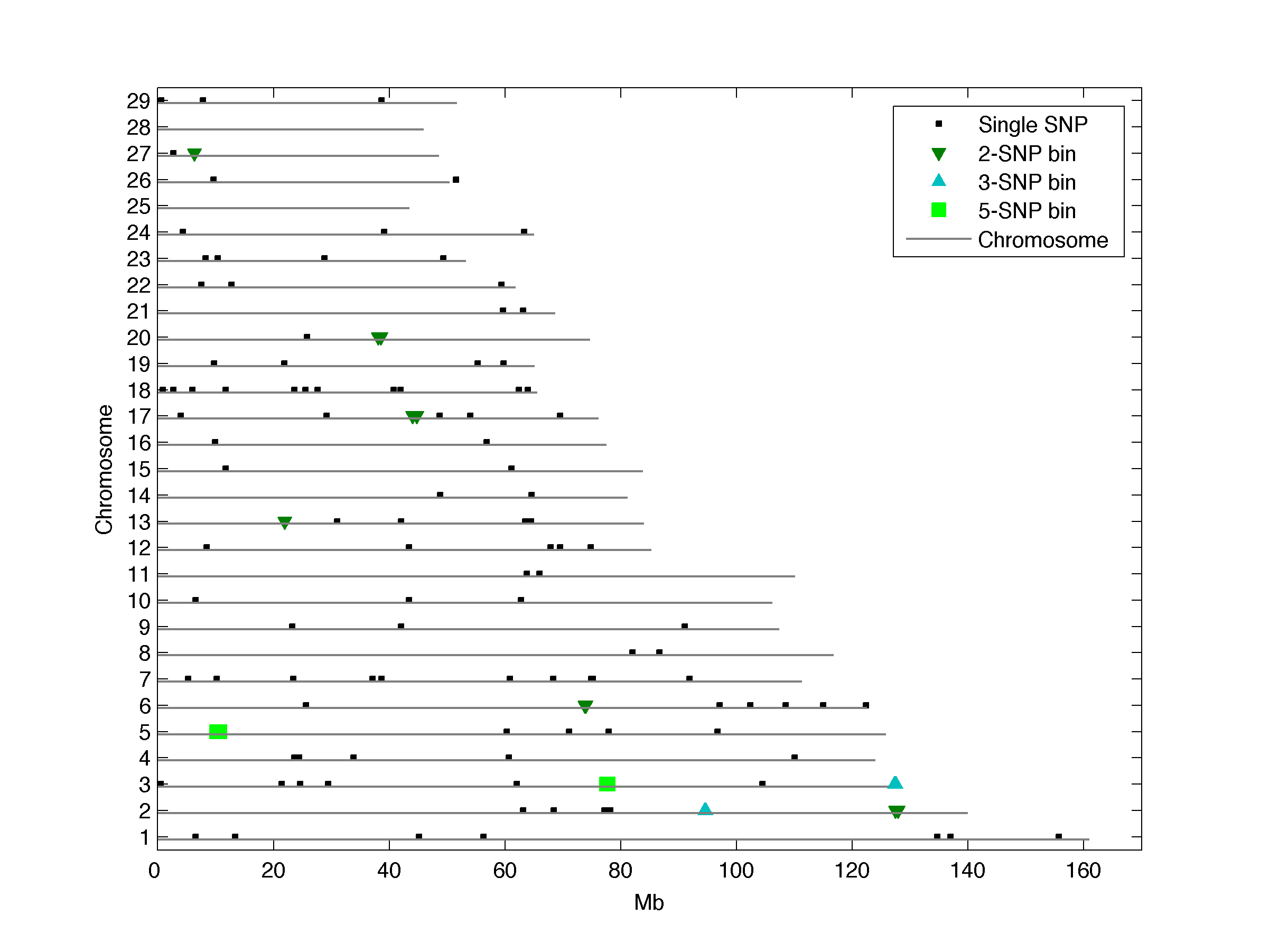

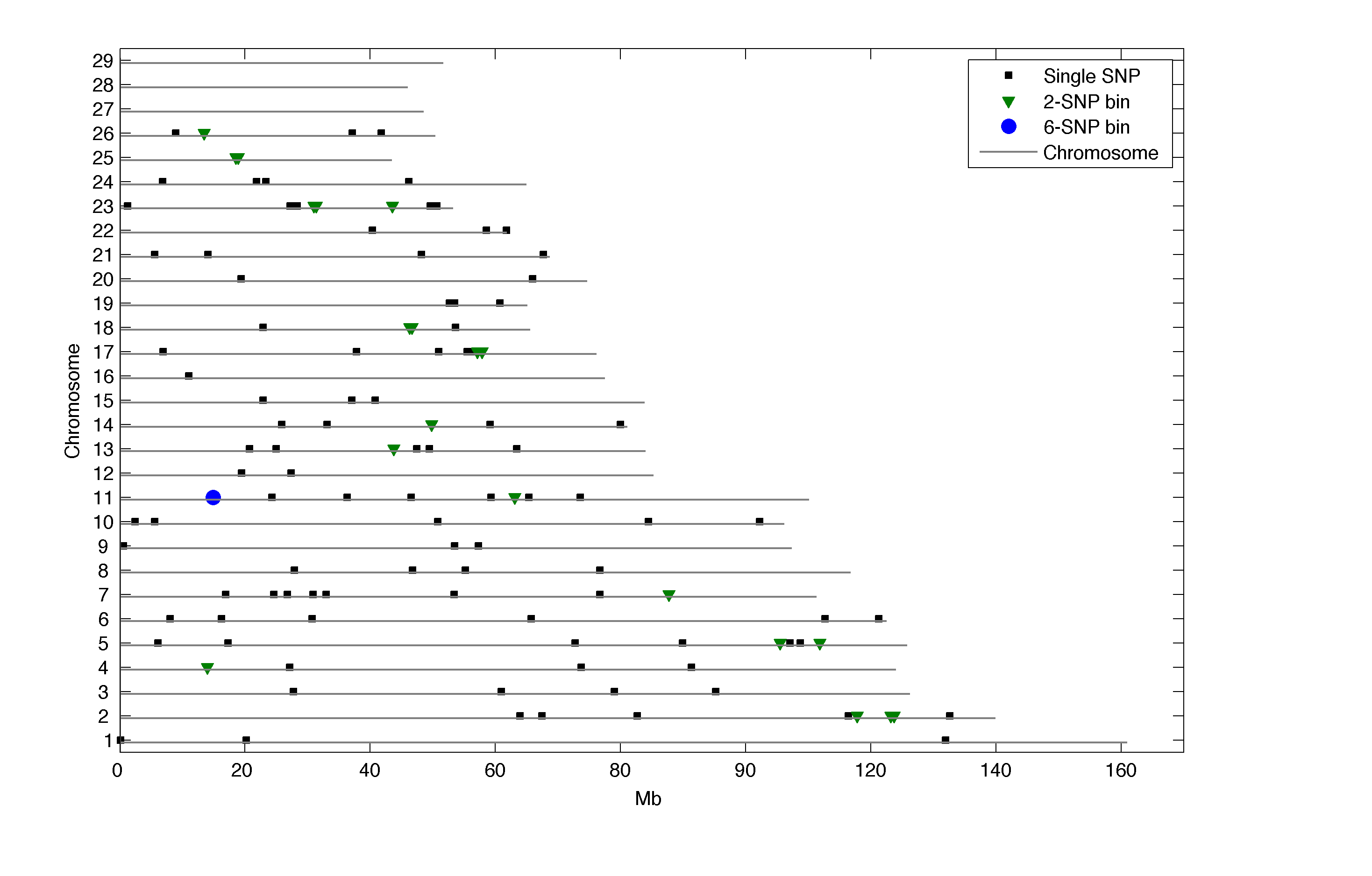
**
