# Supplementary material for: Bos taurus genome assembly
Source: BMC Genomics. 2009 Apr 24;10:180. doi: 10.1186/1471-2164-10-180 (PMC2686734; doi:10.1186/1471-2164-10-180)
Supplement: Additional file 4 — SNPs with linkage position different from Btau_4.0 assembly postion. Table provides list of SNPs with linkage positions that disagree with Btau_4.0 assembly, also provides the identity and position of the most closely linked SNP. Columns include SNP name, chromosome, position in Btau_4.0, and best two-point hit, with the chromosome and position for that linked SNP. [file 1471-2164-10-180-S4.doc]

**Additional file 4. SNPs with linkage position different from Btau_4.0 assembly postion.**

| **SNPs whose linkage position disagrees with Btau_4.0 assembly** | | | **Identity and position of marker most closely linked to misplaced SNP** | | |
| --- | --- | --- | --- | --- | --- |
| **SNP Name** | **Chromosome** | **Position (bp)** | **Best two-point hit** | **Chromosome** | **Position (bp)** |
| BTA-91058 | Chr03 | 496,870 | BTA-101873 | Chr01 | 113,641 |
| BTA-22932 | Chr05 | 96,688,389 | BTA-39453 | Chr01 | 20,193,913 |
| BTA-35326 | Chr14 | 64,605,641 | rs29011172 | Chr02 | 63,947,669 |
| BTA-94821 | Chr18 | 23,630,972 | rs29021721 | Chr02 | 82,664,319 |
| BTA-94838 | Chr03 | 77,532,870 | rs29012554 | Chr02 | 116,411,308 |
| BTA-94832 | Chr03 | 77,635,639 | rs29019662 | Chr02 | 117,878,624 |
| BTA-98418 | Chr03 | 77,714,539 | rs29019662 | Chr02 | 117,878,624 |
| BTA-98425 | Chr03 | 77,859,105 | BTA-99198 | Chr02 | 123,214,857 |
| rs29027394 | Chr03 | 77,719,948 | BTA-49510 | Chr02 | 123,727,852 |
| BTA-50486 | Chr20 | 38,536,865 | rs29017364 | Chr02 | 132,630,739 |
| BTA-26336 | Chr09 | 91,064,769 | BTA-66998 | Chr03 | 27,737,366 |
| rs29020514 | Chr04 | 24,454,416 | BTA-67972 | Chr03 | 60,995,644 |
| BTA-26797 | Chr09 | 23,202,962 | rs29027388 | Chr03 | 79,051,740 |
| BTA-79531 | Chr07 | 60,836,776 | rs29019550 | Chr04 | 14,031,272 |
| BTA-91717 | Chr22 | 59,427,573 | rs29019550 | Chr04 | 14,031,272 |
| BTA-89371 | Chr24 | 63,357,833 | BTA-107052 | Chr04 | 27,108,524 |
| BTA-109198 | Chr10 | 62,741,080 | BTA-99419 | Chr04 | 73,717,948 |
| BTA-04379 | Chr15 | 11,745,818 | rs29015911 | Chr05 | 6,063,005 |
| BTA-109173 | Chr03 | 29,440,968 | rs29010308 | Chr05 | 17,272,101 |
| rs29010446 | Chr10 | 43,390,124 | BTA-74410 | Chr05 | 89,951,148 |
| BTA-74668 | Chr01 | 155,734,273 | rs29022979 | Chr05 | 105,562,352 |
| rs29011109 | Chr02 | 78,243,260 | rs29011106 | Chr06 | 8,032,597 |
| BTA-42607 | Chr18 | 2,721,001 | rs29026830 | Chr06 | 112,708,393 |
| rs29013379 | Chr03 | 24,626,539 | rs29016161 | Chr07 | 24,604,791 |
| rs29019771 | Chr01 | 134,730,386 | rs29027355 | Chr07 | 30,837,807 |
| BTA-20541 | Chr16 | 9,871,322 | rs29014942 | Chr07 | 87,784,874 |
| rs29025385 | Chr14 | 48,778,042 | BTA-29826 | Chr08 | 27,851,464 |
| BTA-88743 | Chr12 | 69,506,929 | ss46526078 | Chr08 | 46,785,406 |
| rs29025267 | Chr18 | 6,007,109 | rs29016119 | Chr08 | 55,229,232 |
| rs29010968 | Chr12 | 74,843,436 | BTA-81828 | Chr08 | 76,689,971 |
| BTA-29912 | Chr13 | 64,462,226 | BTA-103209 | Chr09 | 576,261 |
| BTA-96578 | Chr20 | 25,774,477 | BTA-107069 | Chr09 | 53,495,053 |
| BTA-101378 | Chr06 | 102,412,108 | BTA-92850 | Chr09 | 57,335,761 |
| BTA-03895 | Chr03 | 127,631,252 | rs29025820 | Chr10 | 5,581,166 |
| BTA-111277 | Chr26 | 9,639,393 | BTA-20737 | Chr10 | 50,804,748 |
| rs29026966 | Chr01 | 137,000,997 | BTA-78306 | Chr10 | 84,473,067 |
| BTA-93238 | Chr04 | 33,806,223 | BTA-82521 | Chr10 | 102,253,006 |
| BTA-120530 | Chr15 | 61,095,156 | BTA-24694 | Chr11 | 14,870,693 |
| BTA-16672 | Chr21 | 63,125,150 | BTA-24694 | Chr11 | 14,870,693 |
| BTA-33179 | Chr13 | 63,523,575 | BTA-24694 | Chr11 | 14,870,693 |
| BTA-44093 | Chr18 | 836,599 | BTA-24694 | Chr11 | 14,870,693 |
| BTA-52807 | Chr21 | 59,669,603 | BTA-24694 | Chr11 | 14,870,693 |
| rs29020772 | Chr08 | 82,043,001 | BTA-24694 | Chr11 | 14,870,693 |
| rs29021132 | Chr06 | 73,999,575 | BTA-119580 | Chr11 | 24,266,649 |
| BTA-89729 | Chr01 | 45,151,037 | BTA-110264 | Chr11 | 36,286,441 |
| BTA-98132 | Chr02 | 94,707,658 | rs29014674 | Chr11 | 59,350,664 |
| BTA-98133 | Chr02 | 94,653,349 | BTA-26188 | Chr11 | 63,102,549 |
| BTA-99111 | Chr02 | 94,496,897 | BTA-26188 | Chr11 | 63,102,549 |
| BTA-58133 | Chr24 | 39,101,157 | rs29018916 | Chr11 | 65,336,525 |
| rs29011347 | Chr29 | 616,680 | BTA-120905 | Chr12 | 19,418,217 |
| BTA-116873 | Chr18 | 62,426,622 | BTA-120912 | Chr12 | 27,398,731 |
| rs29025452 | Chr06 | 122,345,282 | rs29023180 | Chr13 | 20,749,118 |
| BTA-55113 | Chr22 | 7,500,620 | rs29010198 | Chr13 | 25,009,393 |
| BTA-44752 | Chr19 | 21,844,696 | ss46526372 | Chr13 | 43,766,433 |
| BTA-89661 | Chr02 | 68,412,658 | ss46526372 | Chr13 | 43,766,433 |
| BTA-32781 | Chr02 | 127,937,481 | BTA-32830 | Chr13 | 49,438,058 |
| rs29027035 | Chr29 | 38,626,817 | rs29014993 | Chr13 | 63,399,110 |
| rs29024582 | Chr16 | 56,813,259 | BTA-34293 | Chr14 | 25,873,079 |
| rs29027211 | Chr17 | 44,757,986 | BTA-122014 | Chr14 | 33,085,806 |
| BTA-53630 | Chr22 | 12,743,292 | rs29012244 | Chr14 | 49,868,613 |
| rs29023105 | Chr20 | 38,138,911 | rs29012244 | Chr14 | 49,868,613 |
| BTA-28023 | Chr12 | 8,417,796 | rs29010230 | Chr14 | 59,149,745 |
| rs29011178 | Chr12 | 43,406,691 | rs29013602 | Chr14 | 79,992,192 |
| BTA-41018 | Chr17 | 48,752,906 | rs29024676 | Chr15 | 22,919,542 |
| rs29027879 | Chr06 | 108,535,543 | ss46527031 | Chr15 | 37,051,043 |
| rs29016971 | Chr07 | 10,136,050 | BTA-36787 | Chr15 | 40,775,629 |
| ss46526206 | Chr04 | 60,696,646 | rs29018392 | Chr16 | 11,016,207 |
| rs29012379 | Chr08 | 86,673,875 | BTA-41034 | Chr17 | 50,989,802 |
| BTA-41133 | Chr29 | 7,827,945 | rs29018289 | Chr17 | 55,527,334 |
| BTA-45181 | Chr01 | 6,539,423 | BTA-41250 | Chr17 | 57,175,022 |
| BTA-82152 | Chr07 | 38,687,896 | BTA-43589 | Chr18 | 46,289,043 |
| BTA-57119 | Chr23 | 8,288,911 | BTA-46280 | Chr19 | 60,716,140 |
| BTA-32288 | Chr13 | 30,967,798 | BTA-49962 | Chr20 | 19,404,643 |
| ss46526669 | Chr05 | 71,073,802 | BTA-51026 | Chr20 | 65,924,904 |
| BTA-64829 | Chr28 | 11,746,149 | BTA-53111 | Chr21 | 5,583,228 |
| BTA-98013 | Chr23 | 49,377,377 | BTA-97987 | Chr21 | 14,041,999 |
| BTA-52461 | Chr03 | 21,411,324 | BTA-09376 | Chr21 | 48,174,745 |
| rs29023326 | Chr12 | 67,917,541 | BTA-121323 | Chr21 | 67,645,338 |
| BTA-17207 | Chr17 | 44,020,506 | BTA-54455 | Chr22 | 40,381,705 |
| BTA-78514 | Chr03 | 127,284,685 | BTA-28287 | Chr22 | 58,581,589 |
| BTA-55062 | Chr03 | 127,539,783 | BTA-55099 | Chr22 | 61,812,267 |
| BTA-116260 | Chr09 | 42,053,078 | BTA-109732 | Chr23 | 1,241,790 |
| BTA-118889 | Chr27 | 6,388,893 | BTA-56087 | Chr23 | 27,196,973 |
| ss46526437 | Chr04 | 110,028,200 | rs29014789 | Chr23 | 28,315,515 |
| BTA-91331 | Chr27 | 6,334,111 | BTA-68784 | Chr23 | 31,013,890 |
| BTA-63833 | Chr28 | 25,547,296 | BTA-76813 | Chr23 | 31,374,887 |
| rs29014021 | Chr02 | 127,508,327 | BTA-35679 | Chr23 | 43,533,035 |
| BTA-104475 | Chr02 | 63,170,412 | rs29011598 | Chr23 | 49,613,492 |
| BTA-94630 | Chr02 | 77,193,713 | BTA-121426 | Chr24 | 21,836,250 |
| ss46526787 | Chr03 | 62,016,482 | BTA-57638 | Chr24 | 23,308,381 |
| BTA-43191 | Chr18 | 40,782,553 | BTA-58247 | Chr24 | 46,152,631 |
| rs29019811 | Chr01 | 13,401,928 | rs29019819 | Chr25 | 18,572,693 |
| ss46526478 | Chr19 | 9,707,016 | rs29021833 | Chr25 | 18,898,214 |
| rs29012266 | Chr07 | 23,455,699 | BTA-119935 | Chr26 | 8,899,883 |
| rs29021150 | Chr13 | 21,941,427 | rs29021152 | Chr26 | 13,452,082 |
| rs29021151 | Chr13 | 21,940,976 | rs29021152 | Chr26 | 13,452,082 |
| BTA-113368 | Chr05 | 60,314,898 | rs29020110 | Chr26 | 37,171,841 |
| BTA-01881 | Chr06 | 25,649,145 | BTA-75651 | Chr06 | 16,231,242 |
| BTA-10383 | Chr07 | 75,206,633 | BTA-99501 | Chr07 | 16,929,254 |
| BTA-105591 | Chr04 | 23,636,598 | BTA-87607 | Chr03 | 95,208,799 |
| BTA-106274 | Chr18 | 27,616,047 | BTA-54717 | Chr17 | 57,897,409 |
| BTA-110005 | Chr11 | 65,955,232 | rs29022294 | Chr11 | 46,585,513 |
| BTA-111486 | Chr26 | 51,524,512 | rs29025349 | Chr24 | 6,821,181 |
| BTA-113341 | Chr07 | 74,922,492 | rs29015017 | Chr07 | 26,749,529 |
| BTA-113435 | Chr07 | 68,363,814 | BTA-23130 | Chr07 | 53,416,094 |
| BTA-118464 | Chr27 | 2,661,884 | rs29025963 | Chr26 | 41,785,126 |
| BTA-121690 | Chr05 | 77,925,779 | BTA-112870 | Chr04 | 91,381,389 |
| BTA-121697 | Chr05 | 10,356,708 | ss46526649 | Chr05 | 105,562,352 |
| BTA-20169 | Chr17 | 69,499,230 | rs29022398 | Chr17 | 37,778,233 |
| BTA-26383 | Chr11 | 63,730,008 | BTA-95156 | Chr10 | 2,417,050 |
| BTA-32671 | Chr13 | 42,024,571 | BTA-120946 | Chr11 | 73,554,246 |
| BTA-40676 | Chr17 | 4,004,885 | BTA-41894 | Chr13 | 47,449,643 |
| BTA-45843 | Chr19 | 55,236,485 | BTA-45798 | Chr18 | 53,610,522 |
| BTA-46132 | Chr19 | 59,798,519 | rs29015132 | Chr19 | 52,672,996 |
| BTA-56137 | Chr23 | 28,788,903 | BTA-35679 | Chr19 | 53,525,493 |
| BTA-58809 | Chr24 | 4,314,165 | BTA-58890 | Chr23 | 50,639,423 |
| BTA-62267 | Chr10 | 6,493,883 | rs29017673 | Chr07 | 87,784,874 |
| BTA-69007 | Chr03 | 104,463,263 | BTA-68647 | Chr02 | 67,451,125 |
| BTA-74760 | Chr05 | 10,164,917 | rs29022979 | Chr05 | 72,724,502 |
| BTA-74776 | Chr05 | 10,445,152 | BTA-121703 | Chr05 | 108,768,798 |
| BTA-74821 | Chr05 | 10,757,744 | BTA-121703 | Chr05 | 111,840,712 |
| BTA-76788 | Chr06 | 73,790,767 | BTA-21297 | Chr06 | 30,713,750 |
| BTA-77374 | Chr06 | 97,106,620 | BTA-78192 | Chr05 | 111,840,712 |
| BTA-77865 | Chr06 | 115,030,644 | BTA-77957 | Chr06 | 65,739,412 |
| BTA-79675 | Chr07 | 5,224,085 | BTA-78404 | Chr06 | 121,284,172 |
| BTA-85542 | Chr17 | 54,000,358 | BTA-41207 | Chr17 | 56,371,097 |
| BTA-86737 | Chr17 | 29,188,027 | rs29020659 | Chr17 | 6,895,347 |
| BTA-88433 | Chr18 | 63,913,290 | BTA-43888 | Chr18 | 46,688,961 |
| rs29020422 | Chr07 | 37,107,750 | rs29024901 | Chr07 | 32,953,542 |
| rs29021917 | Chr18 | 41,937,656 | BTA-43620 | Chr18 | 22,887,936 |
| rs29023651 | Chr07 | 91,873,789 | rs29014942 | Chr07 | 76,690,558 |
| rs29025363 | Chr23 | 10,365,477 | rs29010781 | Chr23 | 43,533,035 |
| ss46526587 | Chr05 | 10,487,471 | rs29027304 | Chr05 | 107,087,307 |
| ss46526705 | Chr01 | 56,271,518 | BTA-51342 | Chr01 | 131,939,498 |
