# Supplementary material for: Bos taurus genome assembly
Source: BMC Genomics. 2009 Apr 24;10:180. doi: 10.1186/1471-2164-10-180 (PMC2686734; doi:10.1186/1471-2164-10-180)

**Additional file 5. Additional SNP placement by linkage analysis.**

The locations 568 SNPs with previously unassigned position in Btau_4.0 whose location was determined by identifying the pairwise comparison between the unknown SNP and all the mapped SNPs that produced the highest LOD score using the *twopoint* option of CRIMAP.


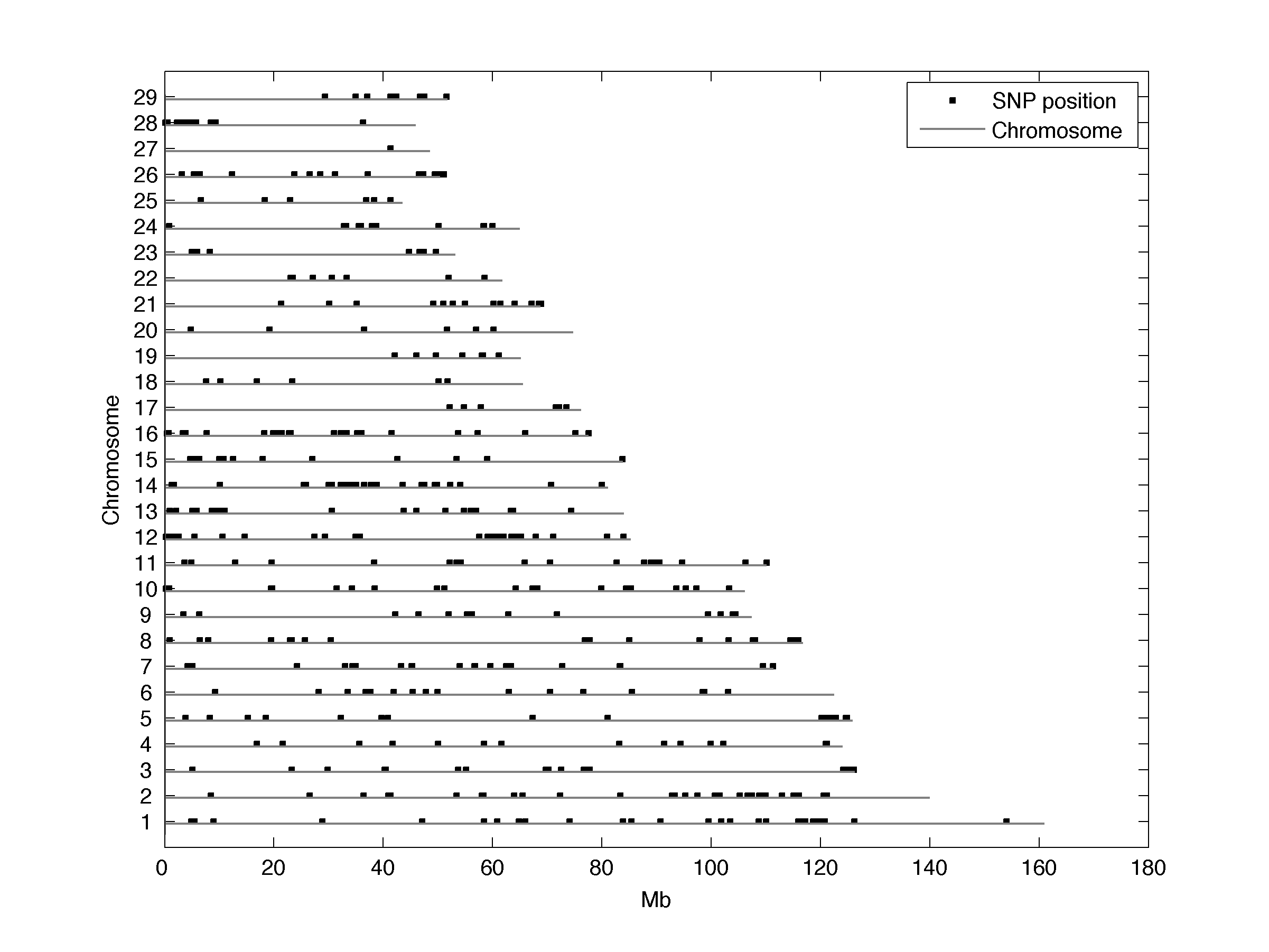

Supplement: Additional file 5 — Additional SNP placement by linkage analysis. The locations of 568 SNPs with previously unassigned position in Btau_4.0 whose location was determined by identifying the pairwise comparison between the unknown SNP and all the mapped SNPs that produced the highest LOD score using the twopoint option of CRIMAP. [file 1471-2164-10-180-S5.doc]
