# Supplementary material for: Bos taurus genome assembly
Source: BMC Genomics. 2009 Apr 24;10:180. doi: 10.1186/1471-2164-10-180 (PMC2686734; doi:10.1186/1471-2164-10-180)
Supplement: Additional file 6 — Placement of unplaced scaffolds using linkage information. Table provides placement information for unplaced scaffolds based on linked markers. Columns include SNP, unplaced Contig, location in unplaced contig, chromosome placement, linked SNP, location in chromosome. [file 1471-2164-10-180-S6.doc]

**Additional file 6. Placement of unplaced scaffolds using linkage information.**

| **Unknown SNP** | **ChrUn Scaffold** | **ChrUn Position** | **Chromosome** | **Best hit SNP** | **Chromosome Position** |
| --- | --- | --- | --- | --- | --- |
| rs29011187 | ChrUn.8448 | 2268 | chr01 | BTA-101248 | 4927996 |
| rs29011186 | ChrUn.8448 | 2149 | chr01 | BTA-35637 | 5485773 |
| rs29011188 | ChrUn.8448 | 2283 | chr01 | BTA-35637 | 5485773 |
| BTA-38481 | ChrUn.900034 | 8740 | chr01 | ss46526282 | 8949725 |
| rs29010971 | ChrUn.768 | 942 | chr01 | BTA-17610 | 28879298 |
| rs29010972 | ChrUn.768 | 912 | chr01 | BTA-17610 | 28879298 |
| BTA-112986 | ChrUn.85 | 325819 | chr01 | rs29010924 | 47153415 |
| rs29016906 | ChrUn.6391 | 2699 | chr01 | BTA-25892 | 58478079 |
| rs29027895 | ChrUn.6391 | 2984 | chr01 | BTA-31648 | 60809664 |
| BTA-108939 | ChrUn.7415 | 558 | chr01 | BTA-92920 | 64787541 |
| BTA-102731 | ChrUn.1001 | 5265 | chr01 | BTA-15416 | 65957389 |
| BTA-36593 | ChrUn.1144 | 19392 | chr01 | BTA-34647 | 74099262 |
| rs29026905 | ChrUn.371 | 122739 | chr01 | BTA-49670 | 83842131 |
| rs29016842 | ChrUn.371 | 28595 | chr01 | BTA-38457 | 85439533 |
| BTA-40479 | ChrUn.793 | 12711 | chr01 | BTA-108728 | 90760334 |
| rs29016980 | ChrUn.321 | 155637 | chr01 | BTA-43342 | 99481432 |
| BTA-24291 | ChrUn.376 | 27030 | chr01 | BTA-45202 | 101800371 |
| BTA-24288 | ChrUn.11 | 403329 | chr01 | rs29026147 | 103459113 |
| BTA-45220 | ChrUn.11 | 1064477 | chr01 | BTA-47181 | 108674076 |
| rs29011084 | ChrUn.11 | 812493 | chr01 | BTA-109427 | 110016500 |
| rs29015912 | ChrUn.1062 | 43953 | chr01 | BTA-89560 | 115969634 |
| BTA-120770 | ChrUn.29 | 602634 | chr01 | rs29013907 | 116820290 |
| BTA-94920 | ChrUn.2067 | 22039 | chr01 | rs29013907 | 116820290 |
| rs29020407 | ChrUn.29 | 175650 | chr01 | rs29013907 | 116820290 |
| rs29017309 | ChrUn.29 | 637696 | chr01 | rs29025427 | 116924173 |
| rs29025995 | ChrUn.747 | 53330 | chr01 | rs29015183 | 116934834 |
| rs29025996 | ChrUn.747 | 53278 | chr01 | rs29015183 | 116934834 |
| rs29025997 | ChrUn.747 | 53216 | chr01 | rs29015183 | 116934834 |
| rs29026000 | ChrUn.747 | 53055 | chr01 | rs29015183 | 116934834 |
| rs29026004 | ChrUn.747 | 49579 | chr01 | rs29015183 | 116934834 |
| rs29021574 | ChrUn.29 | 429163 | chr01 | rs29016850 | 117145796 |
| rs29026001 | ChrUn.747 | 53001 | chr01 | BTA-108007 | 117242907 |
| rs29026857 | ChrUn.558 | 11958 | chr01 | BTA-98510 | 118692414 |
| BTA-106262 | ChrUn.29 | 201881 | chr01 | BTA-49345 | 119440107 |
| rs29019318 | ChrUn.29 | 82462 | chr01 | rs29027928 | 119794066 |
| rs29019317 | ChrUn.29 | 82131 | chr01 | ss46526795 | 120762670 |
| BTA-22054 | ChrUn.973 | 16119 | chr01 | BTA-117044 | 126236416 |
| rs29021139 | ChrUn.1404 | 17493 | chr01 | BTA-58260 | 154090030 |
| rs29021140 | ChrUn.1404 | 17582 | chr01 | BTA-58260 | 154090030 |
| BTA-44464 | ChrUn.6122 | 1069 | chr02 | BTA-85695 | 8501994 |
| BTA-71797 | ChrUn.5351 | 5779 | chr02 | BTA-85695 | 8501994 |
| BTA-46812 | ChrUn.5898 | 4217 | chr02 | BTA-46822 | 26504945 |
| rs29015345 | ChrUn.766 | 4175 | chr02 | rs29025789 | 36426908 |
| BTA-114830 | ChrUn.765 | 56232 | chr02 | BTA-113480 | 40975539 |
| BTA-19863 | ChrUn.91 | 43891 | chr02 | rs29025956 | 41323449 |
| rs29018910 | ChrUn.297 | 11572 | chr02 | BTA-47615 | 53417961 |
| rs29012315 | ChrUn.297 | 37711 | chr02 | BTA-81617 | 58063626 |
| rs29022260 | ChrUn.1449 | 5597 | chr02 | BTA-101346 | 58249620 |
| rs29019511 | ChrUn.1662 | 21710 | chr02 | BTA-101347 | 58366502 |
| rs29027656 | ChrUn.490 | 88733 | chr02 | rs29011172 | 63947669 |
| rs29022259 | ChrUn.1449 | 6045 | chr02 | rs29015936 | 65535887 |
| BTA-68746 | ChrUn.853 | 26146 | chr02 | BTA-48003 | 72394455 |
| rs29015148 | ChrUn.978 | 785 | chr02 | BTA-86183 | 83335055 |
| rs29015149 | ChrUn.978 | 899 | chr02 | BTA-86183 | 83335055 |
| rs29023747 | ChrUn.3074 | 3455 | chr02 | BTA-93779 | 92842235 |
| BTA-120370 | ChrUn.382 | 96706 | chr02 | BTA-100127 | 93320532 |
| rs29021837 | ChrUn.3457 | 6675 | chr02 | rs29018841 | 95276319 |
| rs29021835 | ChrUn.3457 | 6879 | chr02 | BTA-96893 | 97495669 |
| rs29021838 | ChrUn.3457 | 6600 | chr02 | BTA-48798 | 100720777 |
| BTA-97780 | ChrUn.3 | 359900 | chr02 | ss46526411 | 101515792 |
| BTA-47192 | ChrUn.3 | 151505 | chr02 | BTA-120600 | 105251560 |
| BTA-48085 | ChrUn.3 | 762750 | chr02 | BTA-114376 | 106674115 |
| ss46526173 | ChrUn.3 | 808368 | chr02 | BTA-114376 | 106674115 |
| BTA-48077 | ChrUn.3 | 638425 | chr02 | BTA-48495 | 107470593 |
| BTA-48538 | ChrUn.3 | 1185812 | chr02 | BTA-48540 | 108823152 |
| BTA-48076 | ChrUn.3 | 597746 | chr02 | BTA-48803 | 109843756 |
| BTA-48601 | ChrUn.3 | 44668 | chr02 | BTA-48806 | 109956378 |
| BTA-97777 | ChrUn.3 | 424410 | chr02 | BTA-48925 | 112990361 |
| BTA-111230 | ChrUn.9 | 103716 | chr02 | BTA-15018 | 115096615 |
| rs29016479 | ChrUn.405 | 27202 | chr02 | rs29025799 | 116100343 |
| rs29011479 | ChrUn.1701 | 31110 | chr02 | rs29025798 | 116100367 |
| rs29014706 | ChrUn.404 | 90632 | chr02 | BTA-106389 | 120578545 |
| rs29014707 | ChrUn.404 | 90845 | chr02 | BTA-106389 | 120578545 |
| BTA-106384 | ChrUn.2117 | 17133 | chr02 | rs29015006 | 121137206 |
| BTA-11126 | ChrUn.5278 | 775 | chr03 | BTA-111633 | 5061438 |
| ss46527114 | ChrUn.934 | 11690 | chr03 | ss46526687 | 23255401 |
| BTA-122683 | ChrUn.374 | 119066 | chr03 | BTA-67085 | 29829426 |
| BTA-117844 | ChrUn.320 | 35598 | chr03 | BTA-121583 | 40256657 |
| BTA-105889 | ChrUn.1472 | 11198 | chr03 | BTA-17459 | 40456981 |
| rs29014629 | ChrUn.5701 | 2391 | chr03 | rs29017531 | 53702310 |
| rs29010076 | ChrUn.618 | 80727 | chr03 | BTA-67956 | 55162809 |
| rs29020483 | ChrUn.1480 | 11765 | chr03 | BTA-105324 | 69718616 |
| rs29020484 | ChrUn.1480 | 11793 | chr03 | BTA-105324 | 69718616 |
| BTA-114951 | ChrUn.250 | 179367 | chr03 | BTA-68018 | 70253327 |
| BTA-119474 | ChrUn.250 | 131225 | chr03 | rs29013886 | 72584896 |
| BTA-87964 | ChrUn.3871 | 2163 | chr03 | BTA-17163 | 76728899 |
| rs29013282 | ChrUn.5148 | 1039 | chr03 | rs29013285 | 76997488 |
| rs29014702 | ChrUn.404 | 92826 | chr03 | rs29022191 | 77799847 |
| rs29014703 | ChrUn.404 | 92706 | chr03 | rs29022191 | 77799847 |
| BTA-69843 | ChrUn.25 | 272275 | chr03 | BTA-69748 | 124219358 |
| BTA-102275 | ChrUn.25 | 213195 | chr03 | BTA-69793 | 124821624 |
| BTA-101500 | ChrUn.2542 | 15176 | chr03 | BTA-69803 | 125182250 |
| rs29012366 | ChrUn.25 | 681593 | chr03 | rs29021928 | 126097658 |
| rs29016151 | ChrUn.25 | 655701 | chr03 | rs29021928 | 126097658 |
| BTA-70862 | ChrUn.6776 | 3884 | chr04 | BTA-85405 | 16886907 |
| BTA-72148 | ChrUn.3881 | 766 | chr04 | BTA-72158 | 21635538 |
| rs29022997 | ChrUn.761 | 52634 | chr04 | BTA-15856 | 35636863 |
| BTA-119973 | ChrUn.982 | 45470 | chr04 | BTA-23629 | 41683742 |
| BTA-70356 | ChrUn.2695 | 6199 | chr04 | BTA-70361 | 50062728 |
| rs29009902 | ChrUn.700 | 10119 | chr04 | rs29026710 | 58473419 |
| BTA-70804 | ChrUn.700 | 48369 | chr04 | BTA-17767 | 61602681 |
| BTA-110976 | ChrUn.1037 | 45019 | chr04 | BTA-101489 | 83205713 |
| BTA-30784 | ChrUn.9 | 1133738 | chr04 | BTA-87607 | 91381389 |
| rs29011008 | ChrUn.712 | 21374 | chr04 | BTA-115746 | 94384244 |
| rs29011005 | ChrUn.712 | 54429 | chr04 | BTA-71773 | 99881861 |
| rs29011006 | ChrUn.712 | 54405 | chr04 | BTA-71773 | 99881861 |
| rs29011007 | ChrUn.712 | 51432 | chr04 | BTA-71773 | 99881861 |
| rs29027868 | ChrUn.712 | 51105 | chr04 | rs29020597 | 102239360 |
| BTA-26046 | ChrUn.3999 | 3500 | chr04 | rs29019697 | 120976229 |
| BTA-28216 | ChrUn.2120 | 20935 | chr04 | BTA-72633 | 121214561 |
| rs29023398 | ChrUn.720 | 20590 | chr05 | rs29026845 | 3837867 |
| rs29024363 | ChrUn.4898 | 780 | chr05 | BTA-111137 | 8237111 |
| rs29018497 | ChrUn.640 | 61575 | chr05 | rs29016096 | 15208558 |
| rs29018498 | ChrUn.640 | 61551 | chr05 | BTA-20421 | 18533364 |
| BTA-29004 | ChrUn.1410 | 25436 | chr05 | BTA-97188 | 32199409 |
| rs29021717 | ChrUn.7786 | 2341 | chr05 | BTA-73262 | 39714310 |
| rs29014406 | ChrUn.625 | 9393 | chr05 | rs29013530 | 40852253 |
| rs29021931 | ChrUn.5356 | 3952 | chr05 | rs29011637 | 67335731 |
| BTA-74087 | ChrUn.7958 | 3093 | chr05 | rs29022791 | 81052921 |
| rs29011129 | ChrUn.46 | 430420 | chr05 | ss46526971 | 120200555 |
| rs29024261 | ChrUn.3039 | 13447 | chr05 | BTA-75177 | 120911371 |
| rs29011060 | ChrUn.46 | 139216 | chr05 | BTA-23984 | 121611140 |
| BTA-75204 | ChrUn.46 | 366100 | chr05 | BTA-23520 | 121767619 |
| BTA-75439 | ChrUn.101 | 87019 | chr05 | BTA-75395 | 122807308 |
| BTA-75461 | ChrUn.152 | 193883 | chr05 | BTA-103220 | 124690330 |
| rs29013383 | ChrUn.101 | 237154 | chr05 | BTA-32258 | 124854397 |
| rs29013384 | ChrUn.101 | 237109 | chr05 | BTA-32258 | 124854397 |
| rs29013386 | ChrUn.101 | 236685 | chr05 | BTA-32258 | 124854397 |
| rs29023707 | ChrUn.1978 | 5647 | chr06 | rs29016229 | 9256262 |
| rs29023708 | ChrUn.1978 | 2997 | chr06 | rs29016229 | 9256262 |
| BTA-106695 | ChrUn.4044 | 5898 | chr06 | rs29013797 | 28147475 |
| rs29020944 | ChrUn.14 | 442324 | chr06 | BTA-75713 | 33498581 |
| BTA-75789 | ChrUn.14 | 330771 | chr06 | rs29026552 | 36786886 |
| BTA-21803 | ChrUn.14 | 752921 | chr06 | BTA-75776 | 37627697 |
| BTA-21842 | ChrUn.14 | 155387 | chr06 | BTA-75900 | 41867013 |
| rs29016982 | ChrUn.688 | 8347 | chr06 | BTA-76111 | 45406895 |
| rs29009844 | ChrUn.900013 | 1897 | chr06 | rs29016265 | 47812157 |
| rs29020030 | ChrUn.2111 | 3127 | chr06 | BTA-115003 | 49972460 |
| BTA-76553 | ChrUn.8085 | 890 | chr06 | rs29026376 | 62962891 |
| BTA-114665 | ChrUn.5204 | 4587 | chr06 | rs29025576 | 70521277 |
| BTA-81807 | ChrUn.2406 | 3802 | chr06 | BTA-76690 | 70559447 |
| BTA-76945 | ChrUn.5720 | 1201 | chr06 | BTA-76841 | 76582807 |
| rs29020591 | ChrUn.318 | 121136 | chr06 | BTA-111699 | 85526228 |
| rs29020592 | ChrUn.318 | 121201 | chr06 | BTA-111699 | 85526228 |
| BTA-77410 | ChrUn.181 | 50080 | chr06 | BTA-29651 | 98421268 |
| BTA-77481 | ChrUn.2168 | 21547 | chr06 | rs29013689 | 98767650 |
| rs29020621 | ChrUn.2540 | 18579 | chr06 | BTA-77535 | 103144848 |
| BTA-88025 | ChrUn.37 | 391418 | chr07 | rs29025834 | 4175981 |
| rs29027655 | ChrUn.490 | 88876 | chr07 | BTA-79613 | 5061379 |
| BTA-88579 | ChrUn.1573 | 13023 | chr07 | BTA-78679 | 24176655 |
| rs29011118 | ChrUn.359 | 3695 | chr07 | BTA-17793 | 32993262 |
| rs29018438 | ChrUn.1125 | 16651 | chr07 | BTA-116192 | 34370352 |
| rs29018439 | ChrUn.1125 | 16900 | chr07 | BTA-116192 | 34370352 |
| BTA-87293 | ChrUn.6008 | 1705 | chr07 | rs29014417 | 34989404 |
| BTA-78943 | ChrUn.2758 | 4711 | chr07 | BTA-106710 | 43289003 |
| BTA-78905 | ChrUn.483 | 44059 | chr07 | BTA-118481 | 45173085 |
| BTA-79101 | ChrUn.2279 | 12303 | chr07 | BTA-21378 | 45250897 |
| rs29024266 | ChrUn.1476 | 18162 | chr07 | BTA-108601 | 53976767 |
| BTA-79448 | ChrUn.7 | 716781 | chr07 | BTA-79423 | 56735107 |
| BTA-79443 | ChrUn.7 | 754322 | chr07 | rs29015334 | 56817697 |
| BTA-79452 | ChrUn.7 | 960814 | chr07 | BTA-106828 | 59555654 |
| rs29025261 | ChrUn.7 | 313491 | chr07 | BTA-104179 | 62486890 |
| BTA-21871 | ChrUn.7 | 186848 | chr07 | BTA-11496 | 63318570 |
| BTA-79438 | ChrUn.7 | 785073 | chr07 | BTA-112958 | 63399182 |
| rs29012507 | ChrUn.1391 | 1949 | chr07 | BTA-112958 | 63399182 |
| rs29012508 | ChrUn.1391 | 2238 | chr07 | BTA-103786 | 72703587 |
| rs29017192 | ChrUn.538 | 80578 | chr07 | BTA-102261 | 83283438 |
| BTA-22444 | ChrUn.538 | 16904 | chr07 | BTA-102259 | 83319043 |
| BTA-22452 | ChrUn.538 | 63486 | chr07 | BTA-22472 | 83347604 |
| BTA-28827 | ChrUn.47 | 17285 | chr07 | BTA-80515 | 109447065 |
| BTA-117460 | ChrUn.137 | 222290 | chr07 | BTA-80657 | 111298393 |
| BTA-98475 | ChrUn.47 | 266871 | chr07 | rs29026749 | 111326857 |
| rs29013997 | ChrUn.47 | 370654 | chr07 | rs29026749 | 111326857 |
| BTA-10594 | ChrUn.2899 | 8691 | chr08 | rs29017545 | 942550 |
| BTA-120289 | ChrUn.7272 | 564 | chr08 | BTA-120282 | 6458793 |
| BTA-81085 | ChrUn.1624 | 10708 | chr08 | BTA-81987 | 7953043 |
| BTA-29448 | ChrUn.1047 | 34573 | chr08 | rs29012369 | 19486652 |
| BTA-29451 | ChrUn.2458 | 16345 | chr08 | BTA-103065 | 22996780 |
| BTA-64576 | ChrUn.5348 | 1892 | chr08 | BTA-80852 | 23290644 |
| BTA-120697 | ChrUn.2252 | 2530 | chr08 | BTA-103192 | 25628680 |
| BTA-80910 | ChrUn.3909 | 11142 | chr08 | BTA-105450 | 30364310 |
| BTA-73586 | ChrUn.646 | 39190 | chr08 | BTA-121857 | 76926851 |
| BTA-81858 | ChrUn.1528 | 34703 | chr08 | BTA-81860 | 77818564 |
| rs29013589 | ChrUn.1112 | 41034 | chr08 | rs29022333 | 85064076 |
| rs29027400 | ChrUn.1769 | 18908 | chr08 | BTA-21089 | 97868552 |
| rs29009604 | ChrUn.2182 | 5750 | chr08 | BTA-100796 | 103185370 |
| rs29009571 | ChrUn.3263 | 490 | chr08 | BTA-82402 | 107599360 |
| ss46526177 | ChrUn.1350 | 12676 | chr08 | rs29026591 | 107991032 |
| BTA-30991 | ChrUn.9 | 579561 | chr08 | BTA-98771 | 114479718 |
| rs29026942 | ChrUn.48 | 20006 | chr08 | rs29027920 | 115096043 |
| BTA-93444 | ChrUn.42 | 243645 | chr08 | rs29024693 | 115402769 |
| BTA-100386 | ChrUn.48 | 188118 | chr08 | rs29019859 | 115907050 |
| BTA-100393 | ChrUn.42 | 491658 | chr08 | rs29019859 | 115907050 |
| BTA-100395 | ChrUn.48 | 261295 | chr08 | rs29023049 | 115941728 |
| ss46526330 | ChrUn.59 | 178322 | chr08 | rs29023049 | 115941728 |
| rs29017610 | ChrUn.219 | 113655 | chr09 | BTA-83363 | 3407306 |
| BTA-85304 | ChrUn.219 | 89712 | chr09 | rs29026616 | 6367774 |
| BTA-83557 | ChrUn.8316 | 2065 | chr09 | BTA-17234 | 42166811 |
| rs29020438 | ChrUn.1361 | 25735 | chr09 | BTA-109443 | 46464058 |
| BTA-86891 | ChrUn.34 | 268595 | chr09 | BTA-83658 | 51986820 |
| rs29026083 | ChrUn.22 | 677723 | chr09 | rs29016141 | 55391845 |
| rs29016306 | ChrUn.310 | 113796 | chr09 | rs29009686 | 56206860 |
| rs29016307 | ChrUn.310 | 113744 | chr09 | rs29009686 | 56206860 |
| BTA-100373 | ChrUn.22 | 654338 | chr09 | BTA-83927 | 62848329 |
| BTA-21100 | ChrUn.22 | 585926 | chr09 | BTA-83927 | 62848329 |
| BTA-84668 | ChrUn.4158 | 5904 | chr09 | BTA-102671 | 71817302 |
| BTA-84825 | ChrUn.4916 | 2983 | chr09 | rs29027271 | 99397801 |
| rs29018064 | ChrUn.324 | 94348 | chr09 | BTA-85042 | 101723746 |
| rs29018066 | ChrUn.324 | 94080 | chr09 | BTA-85042 | 101723746 |
| rs29018067 | ChrUn.324 | 93936 | chr09 | BTA-85042 | 101723746 |
| rs29020464 | ChrUn.1768 | 15278 | chr09 | BTA-85042 | 101723746 |
| rs29020465 | ChrUn.1768 | 15534 | chr09 | BTA-85042 | 101723746 |
| rs29018065 | ChrUn.324 | 94122 | chr09 | BTA-85167 | 103940109 |
| BTA-85133 | ChrUn.324 | 133493 | chr09 | BTA-97562 | 104462689 |
| rs29018535 | ChrUn.360 | 13828 | chr10 | rs29018536 | 234438 |
| rs29012694 | ChrUn.269 | 81535 | chr10 | BTA-17734 | 813449 |
| BTA-60312 | ChrUn.5066 | 7962 | chr10 | BTA-59356 | 19460999 |
| BTA-59382 | ChrUn.1555 | 748 | chr10 | BTA-59376 | 19694375 |
| BTA-63294 | ChrUn.4527 | 942 | chr10 | rs29019500 | 31457482 |
| BTA-105621 | ChrUn.5411 | 1322 | chr10 | rs29015924 | 34285266 |
| BTA-75457 | ChrUn.56 | 211541 | chr10 | BTA-111218 | 38408712 |
| BTA-68241 | ChrUn.3037 | 2905 | chr10 | rs29013467 | 49803491 |
| BTA-70184 | ChrUn.519 | 2972 | chr10 | rs29025476 | 51216078 |
| BTA-47175 | ChrUn.50 | 69491 | chr10 | BTA-73316 | 64261015 |
| BTA-98725 | ChrUn.6269 | 1020 | chr10 | rs29019194 | 67310551 |
| rs29017533 | ChrUn.6269 | 493 | chr10 | BTA-100671 | 68247675 |
| rs29020679 | ChrUn.1250 | 30125 | chr10 | BTA-86239 | 79864903 |
| BTA-30851 | ChrUn.9 | 817825 | chr10 | BTA-78306 | 84473067 |
| rs29016350 | ChrUn.1193 | 35221 | chr10 | BTA-78321 | 85356289 |
| rs29021103 | ChrUn.499 | 88843 | chr10 | BTA-80377 | 93622262 |
| BTA-100883 | ChrUn.114 | 20729 | chr10 | rs29010022 | 95372032 |
| rs29021108 | ChrUn.499 | 88627 | chr10 | BTA-81356 | 97279266 |
| rs29019517 | ChrUn.1844 | 23534 | chr10 | BTA-109684 | 103295043 |
| rs29027573 | ChrUn.1919 | 22928 | chr11 | BTA-93090 | 3612595 |
| BTA-98053 | ChrUn.705 | 46815 | chr11 | BTA-89411 | 4838605 |
| rs29014587 | ChrUn.704 | 13838 | chr11 | BTA-85479 | 12891182 |
| rs29014588 | ChrUn.704 | 14154 | chr11 | BTA-85479 | 12891182 |
| rs29021569 | ChrUn.5048 | 7862 | chr11 | rs29021573 | 19530290 |
| BTA-91968 | ChrUn.7029 | 3572 | chr11 | rs29011720 | 38286907 |
| BTA-111293 | ChrUn.4129 | 4215 | chr11 | BTA-111292 | 52184674 |
| BTA-97065 | ChrUn.6753 | 4781 | chr11 | rs29014922 | 53413825 |
| rs29013809 | ChrUn.1498 | 22963 | chr11 | rs29014922 | 53413825 |
| rs29013810 | ChrUn.1498 | 22809 | chr11 | BTA-98048 | 54192613 |
| BTA-100183 | ChrUn.475 | 25337 | chr11 | BTA-113290 | 65893071 |
| rs29015810 | ChrUn.6758 | 3393 | chr11 | BTA-101102 | 70562084 |
| rs29018198 | ChrUn.5102 | 4922 | chr11 | rs29011510 | 82691056 |
| rs29011486 | ChrUn.3581 | 11894 | chr11 | rs29012044 | 87730076 |
| rs29015777 | ChrUn.4462 | 5881 | chr11 | BTA-109346 | 89018683 |
| rs29011481 | ChrUn.3581 | 12044 | chr11 | rs29017416 | 89954541 |
| rs29011487 | ChrUn.3581 | 12016 | chr11 | rs29017416 | 89954541 |
| rs29023233 | ChrUn.3581 | 7446 | chr11 | rs29017416 | 89954541 |
| rs29023234 | ChrUn.3581 | 7574 | chr11 | rs29017416 | 89954541 |
| rs29023239 | ChrUn.3581 | 7958 | chr11 | rs29017416 | 89954541 |
| BTA-111376 | ChrUn.4068 | 921 | chr11 | BTA-109362 | 90195905 |
| BTA-111382 | ChrUn.5179 | 7034 | chr11 | rs29012479 | 90504201 |
| rs29014606 | ChrUn.3164 | 9010 | chr11 | BTA-113548 | 94722393 |
| BTA-30999 | ChrUn.9 | 428615 | chr11 | rs29018426 | 106321324 |
| BTA-15480 | ChrUn.135 | 91382 | chr11 | BTA-69399 | 110120453 |
| BTA-117766 | ChrUn.4 | 909683 | chr12 | BTA-122265 | 219151 |
| BTA-17360 | ChrUn.4 | 342039 | chr12 | BTA-27508 | 849971 |
| BTA-18462 | ChrUn.4 | 1373004 | chr12 | BTA-93283 | 1315666 |
| rs29023482 | ChrUn.745 | 4950 | chr12 | BTA-87231 | 1890850 |
| rs29023483 | ChrUn.745 | 4968 | chr12 | BTA-87231 | 1890850 |
| BTA-117763 | ChrUn.4 | 785716 | chr12 | BTA-87236 | 1935386 |
| BTA-25462 | ChrUn.4 | 750076 | chr12 | BTA-87243 | 2360301 |
| BTA-87346 | ChrUn.4 | 1561676 | chr12 | BTA-15663 | 2566754 |
| rs29025374 | ChrUn.4 | 1422374 | chr12 | BTA-18534 | 5442116 |
| BTA-29132 | ChrUn.4 | 1299779 | chr12 | BTA-89932 | 10539394 |
| BTA-31727 | ChrUn.1406 | 771 | chr12 | rs29024514 | 14605515 |
| rs29019945 | ChrUn.124 | 263166 | chr12 | BTA-120912 | 27398731 |
| ss46526440 | ChrUn.992 | 54859 | chr12 | BTA-120912 | 27398731 |
| BTA-96400 | ChrUn.2425 | 15981 | chr12 | BTA-83051 | 29298576 |
| BTA-21636 | ChrUn.890 | 3717 | chr12 | BTA-120916 | 34951640 |
| BTA-116793 | ChrUn.1654 | 3277 | chr12 | BTA-21639 | 35745307 |
| BTA-115965 | ChrUn.23 | 267620 | chr12 | BTA-101760 | 57534765 |
| BTA-29617 | ChrUn.51 | 424842 | chr12 | rs29021135 | 59088156 |
| BTA-26814 | ChrUn.23 | 642617 | chr12 | rs29021621 | 59613637 |
| rs29018670 | ChrUn.203 | 151235 | chr12 | BTA-109586 | 59971853 |
| rs29018674 | ChrUn.203 | 151591 | chr12 | BTA-109586 | 59971853 |
| rs29018675 | ChrUn.203 | 151638 | chr12 | BTA-109586 | 59971853 |
| BTA-16641 | ChrUn.83 | 188735 | chr12 | BTA-66741 | 60418945 |
| BTA-17042 | ChrUn.23 | 89821 | chr12 | rs29017619 | 61187741 |
| BTA-19440 | ChrUn.427 | 98697 | chr12 | rs29017619 | 61187741 |
| BTA-99856 | ChrUn.83 | 130816 | chr12 | BTA-89179 | 61303731 |
| BTA-16633 | ChrUn.51 | 1307 | chr12 | BTA-68851 | 61882021 |
| rs29018449 | ChrUn.83 | 375745 | chr12 | BTA-68851 | 61882021 |
| rs29018671 | ChrUn.203 | 151267 | chr12 | BTA-101786 | 62041477 |
| rs29018673 | ChrUn.203 | 151466 | chr12 | BTA-101786 | 62041477 |
| BTA-16537 | ChrUn.51 | 63345 | chr12 | BTA-27003 | 63440477 |
| rs29016506 | ChrUn.83 | 34347 | chr12 | BTA-27003 | 63440477 |
| BTA-26811 | ChrUn.23 | 679778 | chr12 | BTA-56423 | 64255448 |
| BTA-29871 | ChrUn.83 | 318374 | chr12 | BTA-122769 | 65168742 |
| BTA-91524 | ChrUn.3637 | 10177 | chr12 | rs29014773 | 67898103 |
| BTA-104944 | ChrUn.5 | 297762 | chr12 | BTA-30008 | 71135620 |
| BTA-31416 | ChrUn.8786 | 1088 | chr12 | BTA-98950 | 80998850 |
| rs29011539 | ChrUn.4530 | 3645 | chr12 | BTA-31489 | 83972425 |
| BTA-106122 | ChrUn.8 | 667213 | chr13 | rs29015580 | 896969 |
| BTA-30520 | ChrUn.6 | 610635 | chr13 | BTA-23123 | 2061464 |
| BTA-95194 | ChrUn.8 | 952124 | chr13 | rs29012013 | 5056004 |
| rs29027000 | ChrUn.372 | 92790 | chr13 | rs29020820 | 5811308 |
| rs29027001 | ChrUn.372 | 92738 | chr13 | rs29020820 | 5811308 |
| rs29012159 | ChrUn.8 | 1002643 | chr13 | BTA-33105 | 8665364 |
| BTA-33317 | ChrUn.8 | 45250 | chr13 | BTA-100870 | 9375005 |
| BTA-95054 | ChrUn.8 | 1046244 | chr13 | BTA-100866 | 9434468 |
| BTA-33438 | ChrUn.8 | 147331 | chr13 | BTA-95193 | 9527617 |
| BTA-33458 | ChrUn.8 | 236484 | chr13 | BTA-95193 | 9527617 |
| rs29022427 | ChrUn.8 | 834653 | chr13 | rs29024864 | 9638217 |
| rs29018903 | ChrUn.1156 | 13899 | chr13 | rs29024863 | 9638299 |
| BTA-33394 | ChrUn.8 | 371793 | chr13 | rs29019454 | 10425355 |
| ss46526494 | ChrUn.8 | 178104 | chr13 | rs29019454 | 10425355 |
| BTA-96650 | ChrUn.8 | 472091 | chr13 | rs29011288 | 10811079 |
| BTA-33535 | ChrUn.8 | 778832 | chr13 | ss46527038 | 11013199 |
| rs29013822 | ChrUn.3218 | 7062 | chr13 | rs29013820 | 30551956 |
| rs29013823 | ChrUn.3218 | 7006 | chr13 | rs29013821 | 30552086 |
| BTA-30796 | ChrUn.9 | 1174478 | chr13 | ss46526372 | 43766433 |
| rs29022199 | ChrUn.3124 | 7149 | chr13 | BTA-32801 | 46102567 |
| rs29020917 | ChrUn.256 | 126283 | chr13 | rs29026734 | 51389627 |
| rs29020921 | ChrUn.256 | 126550 | chr13 | rs29026734 | 51389627 |
| rs29020922 | ChrUn.256 | 126684 | chr13 | rs29026734 | 51389627 |
| BTA-115028 | ChrUn.256 | 30805 | chr13 | BTA-32907 | 54742218 |
| rs29020918 | ChrUn.256 | 129342 | chr13 | BTA-32907 | 54742218 |
| rs29020920 | ChrUn.256 | 128841 | chr13 | BTA-32907 | 54742218 |
| BTA-115016 | ChrUn.256 | 140725 | chr13 | BTA-32944 | 55978619 |
| rs29025257 | ChrUn.256 | 27740 | chr13 | BTA-33033 | 56944692 |
| rs29027540 | ChrUn.9 | 341278 | chr13 | rs29014994 | 63399258 |
| rs29010938 | ChrUn.288 | 30135 | chr13 | BTA-33163 | 63708121 |
| BTA-33698 | ChrUn.1967 | 6442 | chr13 | BTA-33838 | 74341206 |
| rs29014765 | ChrUn.3650 | 2847 | chr13 | BTA-33838 | 74341206 |
| BTA-35974 | ChrUn.7314 | 2994 | chr14 | BTA-25132 | 1288297 |
| BTA-34302 | ChrUn.209 | 205525 | chr14 | rs29012138 | 1647400 |
| rs29024687 | ChrUn.209 | 115748 | chr14 | rs29012138 | 1647400 |
| BTA-57149 | ChrUn.6668 | 1908 | chr14 | rs29021170 | 10106926 |
| BTA-92751 | ChrUn.1 | 466425 | chr14 | rs29011291 | 25484635 |
| BTA-107889 | ChrUn.1 | 2714530 | chr14 | rs29027563 | 25879096 |
| rs29023098 | ChrUn.1 | 3521583 | chr14 | rs29027563 | 25879096 |
| BTA-107708 | ChrUn.1 | 2989358 | chr14 | BTA-44398 | 30038418 |
| BTA-107905 | ChrUn.1 | 2261094 | chr14 | BTA-44398 | 30038418 |
| BTA-34690 | ChrUn.1 | 1073875 | chr14 | BTA-34536 | 30580221 |
| rs29012556 | ChrUn.1 | 1039927 | chr14 | BTA-34536 | 30580221 |
| rs29025705 | ChrUn.1 | 2772173 | chr14 | BTA-34536 | 30580221 |
| rs29025708 | ChrUn.1 | 2771882 | chr14 | BTA-34536 | 30580221 |
| BTA-113824 | ChrUn.1 | 732694 | chr14 | BTA-40164 | 32205805 |
| BTA-34668 | ChrUn.1 | 1356370 | chr14 | BTA-40164 | 32205805 |
| BTA-34684 | ChrUn.1 | 1767131 | chr14 | BTA-40164 | 32205805 |
| rs29011659 | ChrUn.1 | 1244412 | chr14 | BTA-40164 | 32205805 |
| ss46527072 | ChrUn.1 | 1352065 | chr14 | BTA-40164 | 32205805 |
| ss46527073 | ChrUn.1 | 1351611 | chr14 | BTA-40164 | 32205805 |
| rs29026742 | ChrUn.1 | 2449929 | chr14 | BTA-105019 | 32421665 |
| rs29018588 | ChrUn.1 | 2166682 | chr14 | BTA-40148 | 32633319 |
| BTA-107705 | ChrUn.1 | 3031128 | chr14 | BTA-122014 | 33085806 |
| BTA-22299 | ChrUn.1 | 2001329 | chr14 | BTA-122014 | 33085806 |
| BTA-34681 | ChrUn.1 | 1693846 | chr14 | BTA-122014 | 33085806 |
| BTA-34689 | ChrUn.1 | 1157913 | chr14 | BTA-122014 | 33085806 |
| BTA-34706 | ChrUn.1 | 842975 | chr14 | BTA-122014 | 33085806 |
| rs29027212 | ChrUn.1 | 1987875 | chr14 | BTA-122014 | 33085806 |
| BTA-107711 | ChrUn.1 | 2917292 | chr14 | rs29017348 | 33608813 |
| BTA-34703 | ChrUn.1 | 951449 | chr14 | rs29017348 | 33608813 |
| BTA-107884 | ChrUn.1 | 2606467 | chr14 | BTA-34558 | 34221879 |
| BTA-107713 | ChrUn.342 | 140412 | chr14 | BTA-34559 | 34267690 |
| BTA-107702 | ChrUn.1 | 3316595 | chr14 | BTA-107716 | 35015122 |
| BTA-107703 | ChrUn.1 | 3171984 | chr14 | BTA-107716 | 35015122 |
| BTA-107888 | ChrUn.1 | 2540826 | chr14 | BTA-107716 | 35015122 |
| BTA-107906 | ChrUn.1 | 2657985 | chr14 | BTA-107716 | 35015122 |
| BTA-16955 | ChrUn.7930 | 3225 | chr14 | BTA-63818 | 36523599 |
| BTA-34677 | ChrUn.1 | 1516211 | chr14 | BTA-63818 | 36523599 |
| rs29012557 | ChrUn.1 | 1039736 | chr14 | BTA-63818 | 36523599 |
| BTA-107730 | ChrUn.1 | 3369378 | chr14 | BTA-110915 | 37795298 |
| BTA-92747 | ChrUn.1 | 572669 | chr14 | BTA-110915 | 37795298 |
| BTA-34704 | ChrUn.1 | 872817 | chr14 | BTA-34746 | 38795076 |
| BTA-26684 | ChrUn.1 | 241762 | chr14 | rs29019816 | 43505663 |
| BTA-89884 | ChrUn.1 | 265182 | chr14 | rs29019816 | 43505663 |
| rs29025828 | ChrUn.402 | 81207 | chr14 | rs29012298 | 47064609 |
| rs29026380 | ChrUn.402 | 27629 | chr14 | BTA-111312 | 47471860 |
| rs29024649 | ChrUn.2705 | 16226 | chr14 | rs29013644 | 49407927 |
| BTA-60192 | ChrUn.2216 | 11941 | chr14 | rs29014125 | 49652936 |
| BTA-31771 | ChrUn.4723 | 449 | chr14 | rs29012244 | 49868613 |
| BTA-58534 | ChrUn.576 | 8091 | chr14 | rs29024333 | 52277482 |
| BTA-58540 | ChrUn.576 | 3635 | chr14 | rs29024333 | 52277482 |
| rs29017263 | ChrUn.587 | 8035 | chr14 | BTA-18230 | 54064881 |
| BTA-97523 | ChrUn.2679 | 1668 | chr14 | rs29023476 | 70750938 |
| rs29011240 | ChrUn.2208 | 15208 | chr14 | rs29013602 | 79992192 |
| rs29013551 | ChrUn.1073 | 35675 | chr15 | rs29020495 | 4707548 |
| rs29011371 | ChrUn.423 | 38566 | chr15 | BTA-99645 | 5727843 |
| BTA-105639 | ChrUn.4655 | 4465 | chr15 | BTA-119599 | 6300045 |
| rs29016010 | ChrUn.384 | 100389 | chr15 | BTA-86760 | 9992401 |
| BTA-29987 | ChrUn.28 | 177773 | chr15 | BTA-37247 | 10819187 |
| BTA-36543 | ChrUn.28 | 458442 | chr15 | rs29011136 | 12534688 |
| BTA-122136 | ChrUn.900012 | 11213 | chr15 | BTA-28976 | 17901271 |
| rs29012459 | ChrUn.2958 | 14686 | chr15 | BTA-16085 | 27029462 |
| rs29019793 | ChrUn.289 | 98751 | chr15 | rs29025337 | 42584302 |
| rs29011239 | ChrUn.2208 | 15201 | chr15 | rs29021089 | 53406836 |
| BTA-25743 | ChrUn.1936 | 19381 | chr15 | BTA-30178 | 59000656 |
| BTA-19568 | ChrUn.65 | 406750 | chr15 | rs29018495 | 83796845 |
| BTA-40340 | ChrUn.6440 | 2065 | chr16 | BTA-38862 | 421728 |
| rs29020729 | ChrUn.323 | 4990 | chr16 | BTA-38570 | 534510 |
| rs29023015 | ChrUn.323 | 63525 | chr16 | BTA-38570 | 534510 |
| rs29022448 | ChrUn.113 | 83457 | chr16 | BTA-38603 | 756271 |
| rs29022449 | ChrUn.113 | 83427 | chr16 | BTA-38603 | 756271 |
| rs29023014 | ChrUn.323 | 63553 | chr16 | BTA-38603 | 756271 |
| rs29018187 | ChrUn.98 | 19817 | chr16 | BTA-38921 | 3374362 |
| BTA-103484 | ChrUn.202 | 23181 | chr16 | ss46526339 | 3730856 |
| ss46526395 | ChrUn.98 | 347670 | chr16 | BTA-39235 | 3766597 |
| rs29018186 | ChrUn.98 | 22012 | chr16 | BTA-39213 | 3794618 |
| rs29013331 | ChrUn.759 | 48977 | chr16 | rs29017620 | 7693115 |
| rs29013332 | ChrUn.759 | 48627 | chr16 | rs29017620 | 7693115 |
| BTA-38151 | ChrUn.2 | 1277925 | chr16 | BTA-40323 | 18185763 |
| BTA-38153 | ChrUn.2 | 1471309 | chr16 | rs29026631 | 19906022 |
| BTA-38182 | ChrUn.2 | 856021 | chr16 | rs29016155 | 20105105 |
| BTA-38156 | ChrUn.2 | 1555514 | chr16 | BTA-40383 | 20432956 |
| BTA-121051 | ChrUn.2 | 1110920 | chr16 | BTA-114777 | 21446487 |
| rs29012611 | ChrUn.2 | 1749514 | chr16 | BTA-114777 | 21446487 |
| rs29014904 | ChrUn.2 | 1213587 | chr16 | BTA-114777 | 21446487 |
| ss46526573 | ChrUn.2 | 1778604 | chr16 | BTA-114777 | 21446487 |
| ss46526574 | ChrUn.2 | 1773093 | chr16 | BTA-114777 | 21446487 |
| rs29024619 | ChrUn.2 | 13695 | chr16 | BTA-38205 | 22728886 |
| BTA-38192 | ChrUn.2 | 377575 | chr16 | rs29019509 | 22962769 |
| BTA-38176 | ChrUn.2 | 34363 | chr16 | BTA-88812 | 23008800 |
| BTA-38857 | ChrUn.3 | 1767904 | chr16 | BTA-01412 | 30983359 |
| rs29017344 | ChrUn.927 | 47252 | chr16 | BTA-38625 | 32278543 |
| rs29017345 | ChrUn.927 | 45384 | chr16 | BTA-38625 | 32278543 |
| BTA-38408 | ChrUn.721 | 44019 | chr16 | BTA-38548 | 33295121 |
| rs29024702 | ChrUn.3 | 1899987 | chr16 | rs29026467 | 35204826 |
| BTA-104990 | ChrUn.3 | 1566399 | chr16 | BTA-38802 | 35484891 |
| BTA-38829 | ChrUn.3 | 2195039 | chr16 | BTA-100038 | 35978574 |
| BTA-38814 | ChrUn.3 | 1427641 | chr16 | rs29023456 | 41483232 |
| rs29014711 | ChrUn.1185 | 16487 | chr16 | BTA-39601 | 53706017 |
| rs29027533 | ChrUn.159 | 17597 | chr16 | BTA-65820 | 57238410 |
| BTA-93850 | ChrUn.3 | 1684127 | chr16 | rs29025309 | 65950413 |
| BTA-39732 | ChrUn.481 | 61540 | chr16 | BTA-40226 | 75179404 |
| BTA-119820 | ChrUn.108 | 334500 | chr16 | BTA-40192 | 77581778 |
| rs29011755 | ChrUn.2826 | 12131 | chr17 | BTA-21911 | 52109868 |
| rs29011756 | ChrUn.2826 | 12069 | chr17 | BTA-21911 | 52109868 |
| rs29014368 | ChrUn.2826 | 12032 | chr17 | BTA-21911 | 52109868 |
| BTA-103531 | ChrUn.492 | 1221 | chr17 | ss46526292 | 54752610 |
| rs29023054 | ChrUn.226 | 76500 | chr17 | BTA-41211 | 57827509 |
| BTA-41836 | ChrUn.428 | 69444 | chr17 | rs29016919 | 71543454 |
| ss46526617 | ChrUn.428 | 54443 | chr17 | BTA-41821 | 71714121 |
| BTA-20175 | ChrUn.1536 | 27162 | chr17 | BTA-41772 | 72151921 |
| BTA-41923 | ChrUn.18 | 19014 | chr17 | BTA-41951 | 73476134 |
| BTA-44346 | ChrUn.6207 | 3666 | chr18 | rs29019800 | 7573680 |
| rs29020809 | ChrUn.794 | 63178 | chr18 | BTA-42315 | 10168415 |
| rs29020810 | ChrUn.794 | 63267 | chr18 | BTA-42315 | 10168415 |
| rs29026837 | ChrUn.660 | 70825 | chr18 | BTA-42612 | 16858980 |
| rs29022179 | ChrUn.354 | 24315 | chr18 | BTA-42917 | 23383717 |
| ss46526371 | ChrUn.732 | 50596 | chr18 | BTA-43739 | 50146867 |
| BTA-30996 | ChrUn.9 | 494900 | chr18 | BTA-34809 | 51796869 |
| BTA-45519 | ChrUn.2172 | 5895 | chr19 | BTA-45458 | 42058156 |
| BTA-45543 | ChrUn.7366 | 994 | chr19 | BTA-121188 | 46027079 |
| BTA-25045 | ChrUn.1674 | 10267 | chr19 | BTA-45625 | 49667188 |
| ss46526430 | ChrUn.706 | 29535 | chr19 | BTA-45878 | 54452705 |
| BTA-46040 | ChrUn.241 | 95120 | chr19 | ss46526398 | 58035657 |
| BTA-46039 | ChrUn.241 | 123926 | chr19 | BTA-46092 | 58272466 |
| BTA-46262 | ChrUn.331 | 93338 | chr19 | BTA-46299 | 61096438 |
| BTA-68711 | ChrUn.1236 | 2784 | chr20 | BTA-68718 | 4745147 |
| rs29018421 | ChrUn.1047 | 17049 | chr20 | BTA-49949 | 19217974 |
| BTA-50526 | ChrUn.1321 | 41076 | chr20 | BTA-50243 | 36458435 |
| rs29012816 | ChrUn.1292 | 24021 | chr20 | BTA-50716 | 51667035 |
| BTA-114653 | ChrUn.9512 | 1897 | chr20 | BTA-50817 | 56965212 |
| rs29019548 | ChrUn.374 | 21506 | chr20 | BTA-50849 | 60203096 |
| rs29018136 | ChrUn.816 | 50280 | chr21 | BTA-103368 | 21334087 |
| rs29016321 | ChrUn.673 | 31849 | chr21 | BTA-27801 | 30105866 |
| rs29020058 | ChrUn.369 | 29593 | chr21 | rs29016828 | 35150014 |
| BTA-94458 | ChrUn.176 | 129954 | chr21 | BTA-113427 | 49157464 |
| ss46527034 | ChrUn.909 | 44028 | chr21 | BTA-110408 | 50972300 |
| rs29023366 | ChrUn.447 | 7653 | chr21 | rs29026956 | 51002612 |
| BTA-52499 | ChrUn.11 | 133794 | chr21 | BTA-116485 | 52757018 |
| rs29023365 | ChrUn.447 | 7344 | chr21 | BTA-115008 | 54915164 |
| BTA-52700 | ChrUn.4020 | 4437 | chr21 | BTA-52789 | 60161285 |
| BTA-52785 | ChrUn.582 | 59572 | chr21 | BTA-119917 | 61410494 |
| rs29011526 | ChrUn.2235 | 3233 | chr21 | BTA-119917 | 61410494 |
| BTA-100477 | ChrUn.177 | 73839 | chr21 | rs29026873 | 64050679 |
| rs29015082 | ChrUn.201 | 191805 | chr21 | BTA-53093 | 67177207 |
| BTA-53191 | ChrUn.201 | 784 | chr21 | BTA-53162 | 68508035 |
| BTA-53197 | ChrUn.177 | 203244 | chr21 | BTA-53162 | 68508035 |
| rs29015081 | ChrUn.201 | 191737 | chr21 | BTA-53152 | 68847581 |
| rs29011494 | ChrUn.886 | 52648 | chr22 | rs29015177 | 23043793 |
| BTA-53817 | ChrUn.285 | 109341 | chr22 | BTA-53826 | 23459973 |
| BTA-79148 | ChrUn.4311 | 7146 | chr22 | rs29024109 | 27075097 |
| rs29011527 | ChrUn.400 | 89716 | chr22 | rs29024109 | 27075097 |
| rs29011532 | ChrUn.400 | 94552 | chr22 | rs29024109 | 27075097 |
| rs29011534 | ChrUn.400 | 94274 | chr22 | rs29024109 | 27075097 |
| BTA-112062 | ChrUn.4278 | 4456 | chr22 | BTA-26846 | 30586036 |
| BTA-79151 | ChrUn.4244 | 10892 | chr22 | rs29011317 | 33341910 |
| BTA-54748 | ChrUn.187 | 83568 | chr22 | BTA-54813 | 51965281 |
| rs29022798 | ChrUn.187 | 92109 | chr22 | BTA-54813 | 51965281 |
| BTA-55012 | ChrUn.422 | 104841 | chr22 | BTA-28287 | 58581589 |
| BTA-103783 | ChrUn.39 | 530215 | chr23 | BTA-119576 | 4971949 |
| BTA-86770 | ChrUn.439 | 10457 | chr23 | BTA-19117 | 5741072 |
| BTA-94938 | ChrUn.39 | 10876 | chr23 | BTA-19117 | 5741072 |
| BTA-94939 | ChrUn.39 | 292616 | chr23 | BTA-106895 | 5910471 |
| ss46526846 | ChrUn.999 | 32175 | chr23 | BTA-57118 | 8251574 |
| BTA-107486 | ChrUn.3112 | 12755 | chr23 | BTA-56932 | 44703443 |
| BTA-56642 | ChrUn.340 | 90184 | chr23 | rs29026860 | 46627945 |
| rs29023504 | ChrUn.1225 | 7811 | chr23 | BTA-29321 | 47444533 |
| rs29016163 | ChrUn.6782 | 5369 | chr23 | rs29011598 | 49613492 |
| rs29016237 | ChrUn.940 | 37992 | chr24 | BTA-58892 | 821972 |
| BTA-57764 | ChrUn.5322 | 4147 | chr24 | rs29020396 | 32814373 |
| rs29016489 | ChrUn.1728 | 1361 | chr24 | BTA-57846 | 33227839 |
| rs29019734 | ChrUn.3807 | 3449 | chr24 | BTA-57935 | 35539889 |
| BTA-58035 | ChrUn.5612 | 2294 | chr24 | BTA-57963 | 35907668 |
| rs29022810 | ChrUn.5506 | 4205 | chr24 | rs29023424 | 37939401 |
| rs29024289 | ChrUn.3014 | 440 | chr24 | BTA-58049 | 38708428 |
| rs29026508 | ChrUn.894 | 44094 | chr24 | BTA-97019 | 50125408 |
| BTA-58398 | ChrUn.4000 | 838 | chr24 | rs29020372 | 58376977 |
| BTA-102567 | ChrUn.2551 | 17008 | chr24 | BTA-58652 | 58396897 |
| rs29015105 | ChrUn.1981 | 2283 | chr24 | rs29010178 | 59961879 |
| rs29026047 | ChrUn.8413 | 2933 | chr25 | BTA-112832 | 6635836 |
| BTA-59097 | ChrUn.2505 | 4249 | chr25 | BTA-59643 | 18354530 |
| rs29015991 | ChrUn.4704 | 9700 | chr25 | rs29015990 | 22943428 |
| rs29015992 | ChrUn.4704 | 9723 | chr25 | rs29015990 | 22943428 |
| BTA-60103 | ChrUn.5408 | 4739 | chr25 | rs29020115 | 36887600 |
| BTA-60237 | ChrUn.242 | 84502 | chr25 | rs29020115 | 36887600 |
| BTA-60244 | ChrUn.242 | 26006 | chr25 | BTA-60118 | 38349638 |
| BTA-60412 | ChrUn.7312 | 1407 | chr25 | BTA-60429 | 41366493 |
| rs29012484 | ChrUn.667 | 67791 | chr26 | rs29020974 | 3119967 |
| rs29012485 | ChrUn.667 | 67840 | chr26 | rs29020974 | 3119967 |
| rs29012488 | ChrUn.667 | 68023 | chr26 | rs29020974 | 3119967 |
| rs29017239 | ChrUn.168 | 185257 | chr26 | rs29011996 | 5340702 |
| BTA-60914 | ChrUn.667 | 25012 | chr26 | BTA-61984 | 6406079 |
| BTA-120361 | ChrUn.134 | 262749 | chr26 | BTA-113104 | 12299204 |
| BTA-91009 | ChrUn.1167 | 22402 | chr26 | BTA-122240 | 23755539 |
| rs29024731 | ChrUn.5556 | 5789 | chr26 | rs29017350 | 26567939 |
| rs29024733 | ChrUn.5556 | 6049 | chr26 | rs29014750 | 28491503 |
| rs29024732 | ChrUn.5556 | 5856 | chr26 | BTA-61103 | 31203668 |
| BTA-30720 | ChrUn.104 | 302936 | chr26 | rs29020110 | 37171841 |
| BTA-61829 | ChrUn.7 | 1282594 | chr26 | BTA-22265 | 46575716 |
| BTA-61819 | ChrUn.377 | 40942 | chr26 | rs29012670 | 47322086 |
| BTA-96863 | ChrUn.240 | 74480 | chr26 | BTA-28372 | 49465313 |
| rs29017076 | ChrUn.240 | 78637 | chr26 | BTA-100715 | 50450210 |
| BTA-96871 | ChrUn.240 | 20984 | chr26 | ss46526062 | 50725836 |
| BTA-25480 | ChrUn.2193 | 14648 | chr26 | BTA-85532 | 51054953 |
| BTA-104367 | ChrUn.30 | 411611 | chr27 | rs29020647 | 41294086 |
| BTA-115925 | ChrUn.156 | 30531 | chr28 | BTA-113989 | 103000 |
| rs29012062 | ChrUn.156 | 56977 | chr28 | BTA-113989 | 103000 |
| BTA-86246 | ChrUn.491 | 54857 | chr28 | BTA-64620 | 500260 |
| rs29012732 | ChrUn.296 | 103703 | chr28 | rs29010186 | 2261935 |
| rs29012063 | ChrUn.156 | 57155 | chr28 | BTA-100914 | 3035821 |
| rs29012064 | ChrUn.156 | 57385 | chr28 | BTA-100914 | 3035821 |
| rs29018314 | ChrUn.276 | 155225 | chr28 | BTA-100914 | 3035821 |
| rs29020507 | ChrUn.665 | 18984 | chr28 | BTA-107346 | 3124529 |
| BTA-108372 | ChrUn.5489 | 3690 | chr28 | rs29014913 | 3220800 |
| rs29020415 | ChrUn.296 | 46586 | chr28 | rs29014913 | 3220800 |
| BTA-116004 | ChrUn.296 | 34564 | chr28 | BTA-108370 | 3317258 |
| rs29017611 | ChrUn.610 | 81496 | chr28 | BTA-108370 | 3317258 |
| rs29022194 | ChrUn.261 | 127324 | chr28 | BTA-108376 | 3460323 |
| rs29022195 | ChrUn.261 | 136770 | chr28 | BTA-88506 | 3760295 |
| rs29012731 | ChrUn.296 | 103285 | chr28 | rs29025682 | 4149609 |
| BTA-108815 | ChrUn.1955 | 8072 | chr28 | BTA-106658 | 5083066 |
| BTA-110538 | ChrUn.791 | 18436 | chr28 | BTA-64604 | 5772594 |
| rs29012733 | ChrUn.296 | 106978 | chr28 | BTA-92198 | 8416191 |
| rs29017616 | ChrUn.156 | 18821 | chr28 | BTA-121532 | 9310728 |
| BTA-64170 | ChrUn.450 | 31701 | chr28 | BTA-64179 | 36295371 |
| BTA-91671 | ChrUn.642 | 40106 | chr29 | BTA-65247 | 29349011 |
| BTA-86632 | ChrUn.432 | 18546 | chr29 | BTA-65589 | 34967060 |
| BTA-65660 | ChrUn.7050 | 4477 | chr29 | BTA-65697 | 37113588 |
| BTA-66041 | ChrUn.65 | 117193 | chr29 | BTA-65697 | 37113588 |
| BTA-105079 | ChrUn.3423 | 6654 | chr29 | BTA-17349 | 41310767 |
| BTA-30842 | ChrUn.9 | 907554 | chr29 | BTA-102307 | 42351796 |
| BTA-65638 | ChrUn.3383 | 2491 | chr29 | rs29025626 | 46748615 |
| BTA-66042 | ChrUn.65 | 24385 | chr29 | rs29017443 | 46824437 |
| BTA-44068 | ChrUn.171 | 44416 | chr29 | BTA-66045 | 47525062 |
| BTA-122070 | ChrUn.137 | 81089 | chr29 | rs29019301 | 51539390 |
| rs29020813 | ChrUn.171 | 198223 | chr29 | rs29019301 | 51539390 |
| rs29020815 | ChrUn.171 | 198096 | chr29 | rs29019301 | 51539390 |
| rs29021674 | ChrUn.137 | 147909 | chr29 | rs29019301 | 51539390 |
| rs29026528 | ChrUn.137 | 171475 | chr29 | rs29019301 | 51539390 |
| ss46526467 | ChrUn.137 | 146961 | chr29 | rs29019301 | 51539390 |
| ss46527003 | ChrUn.163 | 72759 | chr29 | rs29019301 | 51539390 |
